# Vitamin B12 Protects Against Early Diabetic Kidney Injury and Alters Clock Gene Expression in Mice

**DOI:** 10.3390/biom15121689

**Published:** 2025-12-03

**Authors:** Niroshani M. W. Wariyapperuma Appuhamillage, Anshulika A. Deshmukh, Rachel L. Moser, Qing Ma, Jiayi Zhou, Feng Li, Yukako Kayashima, Nobuyo Maeda

**Affiliations:** Department of Pathology and Laboratory Medicine, University of North Carolina at Chapel Hill, Chapel Hill, NC 27599, USA; anshulik@live.unc.edu (A.A.D.); mrache@unc.edu (R.L.M.); maqing@email.unc.edu (Q.M.); jz0105@email.unc.edu (J.Z.); feng_li@med.unc.edu (F.L.); yukaya@email.unc.edu (Y.K.); nobuyo@med.unc.edu (N.M.)

**Keywords:** Vitamin B12, circadian rhythm, diabetic nephropathy

## Abstract

Vitamin B12 (B12) is a strong antioxidant and a cofactor for methionine synthase supporting DNA/RNA/protein methylation. We previously demonstrated that oral high-dose B12 supplement mitigates diabetic cardiomyopathy in Akita diabetic mice expressing twice the normal levels of Elmo1 (Engulfment and cell motility 1). To assess how B12 prevents early kidney damage, we treated *Elmo1^HH^* mice and diabetic *Elmo1^HH^ Ins2^Akita/+^* mice with or without B12 in drinking water starting at 8 weeks of age. At 16 weeks, markedly reduced mesangial expansion was detected in the B12-treated diabetic kidneys (22% of glomeruli affected vs. 70% in the untreated diabetic kidneys). RNAseq analysis of the kidneys revealed that B12 suppressed expression of genes for adaptive immune response, while it upregulated those for solute carrier transporters and antioxidant genes. Strikingly, B12 treatment suppressed activators of circadian rhythm, *Clock* and *Bmal1*, and upregulated repressors like *Cry1/2*, *Per1-3* and *Dbp*, suggesting a shift in their rhythmicity. B12 also upregulated linker histone H1 variants, and enhanced chromatin stability and cell cycle regulation. In BU.MPT proximal tubular cells in culture, B12 shifted forward the circadian expression phase of *Bmal1* and *Per1*. Taken together, B12 supplement effectively mitigates early development of diabetic nephropathy in diabetic mice, potentially involving regulation of circadian rhythm.

## 1. Introduction

Vitamin B12 (cobalamin, B12), the largest known vitamin, with a molecular weight of 1355 Da, is an essential micronutrient required for cellular metabolism, DNA synthesis, and red blood cell formation [1]. Because eukaryotes cannot synthesize B12 endogenously, humans and mice must obtain Vitamin B12 from dietary sources, although the average daily requirement is less than 5 µg for humans [2]. Vitamin B12 possesses antioxidant properties, particularly since its cobalt (Co^2+^) center acts as a superoxide scavenger with a reactivity rate comparable to that of superoxide dismutase (SOD) [3]. Oxidative stress is a key contributor to the pathogenesis of diabetic complications, particularly in diabetic nephropathy (DN) [4]. Excessive production of reactive oxygen species (ROS) has been shown to exacerbate tissue damage and inflammation in DN [5,6,7].

Engulfment and Cell Motility 1 (Elmo1) interacts with Dock proteins (Dedicator of cytokinesis), activating Rac1-GTPase. This process is essential for phagocytosis and cell migration [6]. Activated Rac1, as an obligatory subunit of NADPH oxidases, increases superoxide production, resulting in cellular oxidative stress [6]. Single-nucleotide polymorphisms (SNPs) in the *ELMO1* gene are associated with increased risk for DN in different populations [8,9]. We previously reported that mice overexpressing *Elmo1* at 200% of normal (*Elmo1^H/H^*) had elevated ROS levels and developed worsened diabetic complications [6]. Despite similar levels of plasma glucose, insulin, and systolic blood pressure across these lines, oxidative stress markers, such as plasma level of lipid peroxides, erythrocyte levels of reduced glutathione (GSH) and plasma levels of transforming growth factor β1 (TGFβ1), progressively worsened with *Elmo1* overexpression [6]. We further reported that treatment with B12 in diabetic *Elmo1^H/H^* mice improved diabetic cardiomyopathy by modulating oxidative stress and the DNMT–SOCS1/3–IGF-1 signaling pathway [10]. Additionally, high-dose Vitamin B12 administration significantly decreased ROS levels and reduced ischemia/reperfusion-induced kidney injuries in mice [3,11]. A 3-year treatment with citicoline and Vitamin B12 eye drops slowed neuro-retinal degeneration and microvascular damage in type 1 diabetic patients with mild retinopathy [12].

Although our team have previously demonstrated the beneficial effects of B12 on diabetic complications [10,11], the precise molecular mechanisms remain largely unknown. We conducted this study to gain a deeper understanding of how B12 supports kidney health under diabetic conditions, particularly during the early stages of DN in mice. We identified the effects of the circadian clock and linker histone H1 mRNA as being among other highly significant effects for which further understanding is required. B12 not only improves metabolic stability but also reinforces chromatin structure and circadian regulation, thereby offering multi-level protection against DN.

## 2. Materials and Methods

### 2.1. Animal Study

All experiments were conducted using male *Elmo1^H/H^* mice on an C57BL/6J genetic background. *Elmo1^H/H^* mice were mated with *Ins2^Akita/+^* mice (Jackson laboratory, Strain: 003548, Bar Harbor, ME, USA). The C57BL/6-Ins2Akita/J model develops early, persistent hyperglycemia due to misfolded proinsulin causing ER stress and progressive β-cell loss. Limited insulin output starting a young age (∼3–4 weeks) results in weight loss (polydipsia/polyuria) independent of diet after 2 months of age [13]. Mice were allocated into four groups: non-diabetic *Elmo1^H/H^Ins2^+/+^* mice and diabetic *Elmo1^H/H^Ins2^Akita/+^* mice supplemented with B12 starting at eight weeks of age for eight weeks at a dose of 10 mg/kg bw/day, administered via the drinking water [10]. Diabetes halves plasma B12 levels, but B12 at this dose normalizes back to the plasma levels of non-diabetic mice [10]. All groups were provided with rodent chow (Select Rodent 50 IF/9F, 5V5M, PicoLab, Richmond, IN, USA) ad libitum throughout the experimental period. All mice were kept under husbandry conditions, conforming to the National Institutes of Health Guideline for Use and Care of Experimental Animals [13].

### 2.2. Systolic Blood Pressure (SBP) Analysis

SBP was measured eight weeks after B12 treatment using the tail-cuff method [11] with BP-2000 SERIES II, Visitec Systems, Waltham, MA, USA. The mean SBP values are the average of the mean values of five days, each consisting of the mean of 30 trials/day [6].

### 2.3. Sample Collection

Sixteen-week-old B12-treated and non-treated mice were fasted for 3–4 h before being sacrificed. Mice were anesthetized via isoflurane (1.5%) inhalation [11], blood was collected via the retro-orbital vein using heparinized capillary tubes and plasma was obtained by centrifuging at 8000× *g* for 15 min. Mice were euthanized by cervical dislocation for tissue collection. The collected tissues were preserved in RNA later (#Am7021, Invitrogen, Thermo Fisher Scientific, Waltham, MA, USA) for RNA isolation and in 4% paraformaldehyde for histological analysis. Plasma and tissues, later stored in RNA, were kept frozen at −20 °C until further analysis.

### 2.4. Plasma Biological Parameters

Plasma glucose and triglycerides were measured with Wako Autokit Glucose (#997-03001, FUJIFILM Wako, Osaka, Japan) and the Triglyceride Assay Kit (#MA-TG, RayBiotech, Peachtree Corners, GA, USA), respectively. Spot urine samples were collected for the analysis of albumin and creatinine. Urinary albumin was measured using a Murine Microalbuminuria ELISA kit (#LS-F39493, LSBio, Shirley, MA, USA). Urinary creatinine was measured using a creatinine test kit (#80350, Crystal Chem, Elk Grove Village, IL, USA).

### 2.5. Histology and Immunofluorescence

Renal tissues were fixed in 4% buffered formalin, paraffin-embedded, sectioned at 5 µm, deparaffinized and dehydrated. Periodic acid–Schiff (PAS) staining was performed using 0.5% periodic acid (#P7875, Sigma-Aldrich, St. Louis, MO, USA), Schiff’s reagent (#3952016, Sigma-Aldrich), and counterstaining with Harris Hematoxylin (#HHS32, Sigma-Aldrich). Masson’s Trichrome staining was carried out using the standard protocol (Center for Musculoskeletal Research, University of Rochester Medical Center). For immunostaining, kidney sections were subjected to antigen retrieval in 10 mM citrate buffer (pH 6.0). Sections were blocked with 10% normal goat serum and 0.1% Bovine Serum Albumin (BSA; #10035) for 1 h at room temperature, followed by overnight incubation at 4 °C with the following primary antibodies: mouse monoclonal anti-CD19 (#HIB19, 1:200; Invitrogen), PE anti-mouse CD45RB (#A11029, 1:200; BioLegend, San Diego, CA, USA), rat anti-CD3 (17A2; #14-0032-82, 1:200; Invitrogen), mouse anti-iNOS (#Ma5-17139, 1:500; Invitrogen), and rat anti-F4/80 (#70076, 1:500; Cell Signaling, Danvers, MA, USA). After three PBS washes, the sections stained with CD19 and CD3 were incubated with Alexa Fluor 488-conjugated goat anti-mouse IgG (#A11037; 1:500; Invitrogen) and Alexa Fluor 594-conjugated goat anti-rat IgG (#A11007; 1:500; Invitrogen) for 1 h at room temperature. Slides were mounted with 4′,6-diamidino-2-phenylindole (DAPI) Fluoromount G (#0100-20; Southern Biotech, Birmingham, AL, USA) and imaged on a fluorescent microscope (Olympus IX70, Olympus Corporation, Tokyo, Japan).

### 2.6. RNA Sequencing and Data Analysis

Total RNA was extracted using the RNeasy Mini Kit (#74106, QIAGEN, Hilden, Germany) according to the manufacturer’s instructions. The library preparation and RNA sequencing analyses were carried out at the High Throughput Genomic Sequencing Facility, University of North Carolina, Chapel Hill, NC, USA. Raw paired-end 50 bp RNA-seq reads were aligned to the mouse reference genome GRCm39 with gene annotations from GENCODE vM35 using the STAR aligner. Alignments were carried out with 12 threads, producing coordinate-sorted BAM files, retaining splice junction information through the supplied GTF file, assigning strand information using intron motifs, and generating gene-level counts with the GeneCounts option. After alignment, transcript assembly and quantification were performed using StringTie, and count matrices were generated with the associated prepDE.py script. The gene-level count matrix was imported into R for downstream analysis with the DESeq2 1.48.x package. Genes with fewer than 100 total counts across all samples were removed prior to normalization. Count data were normalized using the median-of-ratios method. Genes with an absolute log2 fold change greater than or equal to 0.5 were considered differentially expressed and were visualized with volcano plots. Significantly altered genes were then compared using heat maps with the whole set of mice using normalized counts. Gene expression data were deposited in the NCBI Gene Expression Omnibus (GEO) database (Accession: GSE306999).

### 2.7. Quantitative Reverse-Transcription Polymerase Chain Reaction (qRT-PCR)

Total RNA was extracted from the tissue with Trizol (15596026, Invitrogen, Thermo Fisher Scientific, Waltham, MA, USA), and qRT-PCR was performed according to the previously published method [14]. Expression levels were expressed as relative fold increases/decreases normalized to the housekeeping gene *Actb*, which was constant between groups. The TaqMan universal PCR master mix (Applied Biosystems, Thermo Fisher Scientific, Waltham, MA, USA) and specific primers were used in a 7500 Real-Time PCR system (30 min at 48 °C, 10 min at 95 °C with 40 cycles of 15 s at 95 °C, and 1 min at 60 °C) for quantification purposes. Individual primers and probe sequences are listed in Table S1.

### 2.8. Cell Culture and Synchronization

BU.MPT cells, a conditionally immortalized mouse proximal tubular epithelial cell line [15], were cultured to confluence in Dulbecco’s Modified Eagle Medium (DMEM; 11995-065, Thermo Fisher Scientific) supplemented with 10% fetal bovine serum (FBS; S12450, Sigma-Aldrich), 100 U/mL penicillin-streptomycin (15140-122, Thermo Fisher Scientific), and 10 U/mL interferon-γ (14777, IFN-γ; Sigma-Aldrich) in 5% CO_2_ at the permissive temperature, 37 °C. When confluent, cells were passaged using 1 mL of 0.25% trypsin-EDTA (15050-065, Thermo Fisher Scientific) per 100 mm dish and seeded at 80,000 cells/well in 12-well plates with or without 0.3 μM B12 (Vitamin B12; V2876, Sigma-Aldrich). Plates with/without B12 were then moved and maintained under non-permissive conditions (39 °C, 5% CO_2_, without IFN-γ). Circadian rhythms were synchronized by treatment with 100 nM dexamethasone (#D4902, Sigma-Aldrich) for 2 h. Cells were then washed with Ca^2+^- and Mg^2+^-free Dulbecco’s phosphate-buffered saline (DPBS; #21-031-CV, Corning, Corning, NY, USA) and replaced with fresh medium. Total RNA was collected every 4 h from 12 to 60 h post-synchronization, and expression of genes was quantified by real-time PCR.

### 2.9. Protein Extraction

BU.MPT cells were seeded at 1.5 × 10^5^ cells per well in 6-well plates with or without 0.3 μM B12 and synchronized as above. Total protein was collected every 8 h from 28 to 60 h. After washing with ice-cold PBS, cells were lysed in RIPA buffer [14] supplemented with protease inhibitor cocktail (A32953, Thermo Fisher Scientific) at 4 °C. Lysates were incubated at 4 °C for 30 min and centrifuged at 12,000× *g* for 5 min at 4 °C. Supernatants were transferred to fresh tubes, and protein concentrations were determined using a bicinchoninic acid (BCA) assay (#23225, Pierce™ BCA Protein Assay Kit, Thermo Fisher Scientific).

### 2.10. Western Blott Assay

Equal amounts of protein (13.5 μg) were loaded to 4–10% pre-cast Mini-PROTEAN TGX gels (#4561095, BIO-RAD, Hercules, CA, USA), and samples were electrophoresed and detected as previously described [14]. Membranes were incubated overnight at 4 °C with primary antibodies against Bmal1 (1:1000, #14020, Cell Signaling) and Per1 (1:1000, #13463-1-AP, Proteintech, Rosemont, IL, USA), followed by a 30 min incubation with HRP-linked anti-rabbit IgG secondary antibody (1:1000, #7074, Cell Signaling). Band intensities were quantified using Fiji ImageJ 1.54p (Wayne Rasband and contributors, National Institutes of Health, Bethesda, MD, USA)), and the resulting values were normalized to β-actin (1:5000, #5125, Cell Signaling).

Statistics and reproducibility: All measurements were taken from biologically distinct samples. Data are expressed as means ± standard errors. A multifactorial ANOVA test was used with the program Pro JMP 17.2.0 (SAS Institute, Cary, NC, USA). Post hoc analyses were performed using the student *t*-test as described in the figure legends. The effects of diabetes and of B12 treatment, as well as their interactions, were assessed using the generalized linear model. The reproducibility including biologically independent sample sizes is stated in each figure legend.

## 3. Results

### 3.1. B12 Treatment Improves Multiple Metabolic and Renal Function Parameters in Diabetic Mice

**Figure 1:**
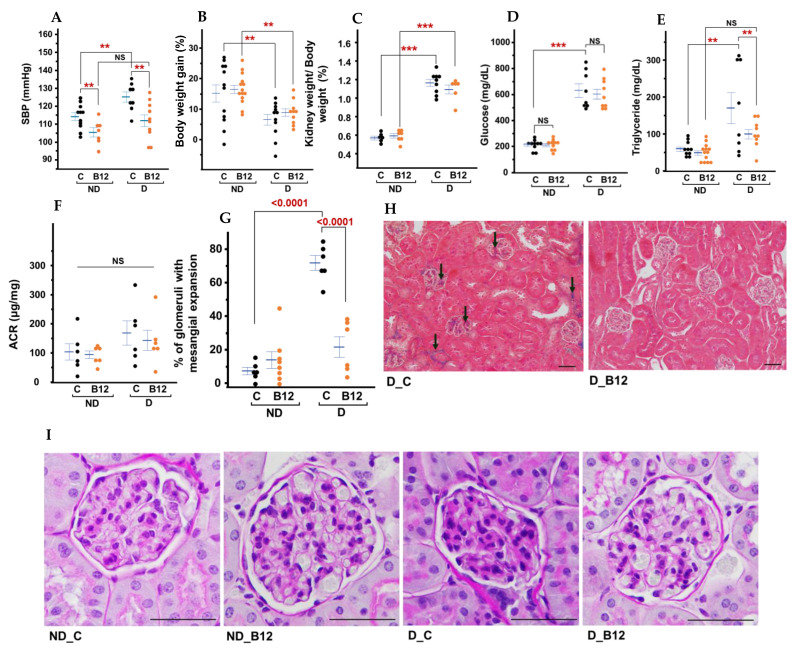
B12 improves pathological and renal parameters in diabetic mice. (**A**) SBP; (**B**) body weight changes from two months to four months of age; (**C**) kidney weight/body weight; (**D**) plasma glucose; (**E**) plasma triglycerides; (**F**) urinary albumin-to-creatinine ratio; (**G**) quantitative assessment of glomerular mesangial matrix expansion performed via blinded histopathological scoring. For each kidney section, 80–100 glomeruli were evaluated. The percentage of glomeruli with mesangial expansion was calculated using the following formula: (number of glomeruli with mesangial expansion/total number of glomeruli analyzed) × 100. (**H**) Masson’s Trichrome staining highlighting fibrosis (black) arrows, Glomerular magnification shown in Figure S1A). (**I**) Periodic acid–Schiff (PAS) staining with hematoxylin of the glomerulus in 16-week-old male mice. Groups: non-diabetic (ND), diabetic (D), control (C), and Vitamin B12-treated (B12). Scale bars: 50 μm. Data are yjr mean ± SEM, *n* = 5–13 mice/group. ** *p* < 0.05, *** *p* < 0.001, NS = not significant.

Systolic blood pressure was elevated in diabetic mice by approximately 10 mmHg, while B12 treatment reduced it to about 10 mmHg in both non-diabetic and diabetic mice (Figure 1A). Body weight gain during the two months of treatment with or without B12 differed markedly between diabetic and non-diabetic groups (Figure 1B). Untreated non-diabetic mice gained ~15% body weight during the two-month treatment period, whereas non-diabetic mice treated with Vitamin B12 gained ~16%. In contrast, untreated diabetic mice showed markedly lower weight gain (~6%). B12 treatment partially attenuated this catabolic effect, resulting in ~9% weight gain (Figure 1B and Table S3). Despite having lower overall body weight, diabetic control mice showed enlarged kidneys, whereas B12-treated diabetic mice exhibited a downward trend in kidney weight, although this was not statistically significant (Figure 1C).

Diabetic mice had higher plasma glucose levels compared to non-diabetic mice, and B12 had no significant effects in either group (Figure 1D). Previous studies have reported similar outcomes, where B12 improved metabolic parameters without fully normalizing hyperglycemia in insulin-deficient models [16]. Given that Akita mice carry a mutation in the *insulin 2* (*Ins2*) gene leading to progressive β-cell apoptosis, the modest glucose-lowering effect observed here is likely secondary to improved peripheral metabolic resilience rather than the direct preservation of β-cell mass. In contrast, a significant improvement in plasma triglyceride levels was observed with B12 treatment, suggesting a role for B12 in lipid metabolism (Figure 1E). In diabetic conditions, triglyceride levels tended to vary widely, but B12 appeared to reduce this variability, indicating a stabilizing effect on lipid metabolism. Additionally, B12 reduced the urine albumin-to-creatinine ratios in both diabetic and non-diabetic mice, but the difference was not statistically significant at this stage (Figure 1F).

At four months of age, kidneys of diabetic mice showed glomerular compression with some extracellular matrix accumulation and fibrosis (Figure 1G–I and Figure S1A). Interstitial fibrosis and peritubular fibrosis were minimal. Structural changes in the control diabetic glomeruli were also evident under hematoxylin and eosin (H&E) staining (Figure S1B). In contrast, kidneys of the B12-treated animals showed normal glomerular morphology with open capillary spaces (Figure 1I). They also exhibited a significantly reduced percentage of glomeruli with mesangial expansion (by ~50%) compared to the untreated diabetic control group (Figure 1G). Masson’s trichrome staining also showed no discernable glomerular fibrosis or peritubular fibrosis following B12 treatment despite diabetic conditions (Figure 1H). Inflammatory cell infiltration was difficult to ascertain in histological sections at this stage. By immunostaining, the presence of CD19-positive B cells was detected in the peritubular space of diabetic kidneys with and without B12 treatment, while naïve T-cell presence appeared more substantial in the B12-supplemented group (Figure S2). Diabetic kidneys showed an increased number of M1 macrophages compared with those under treatment, and this was reduced in B12-treated diabetic mice (Figure S3).

### 3.2. Global Gene Expression Analyses Revealed Pathways Through Which B12 Mitigates DN Development

**Figure 2:**
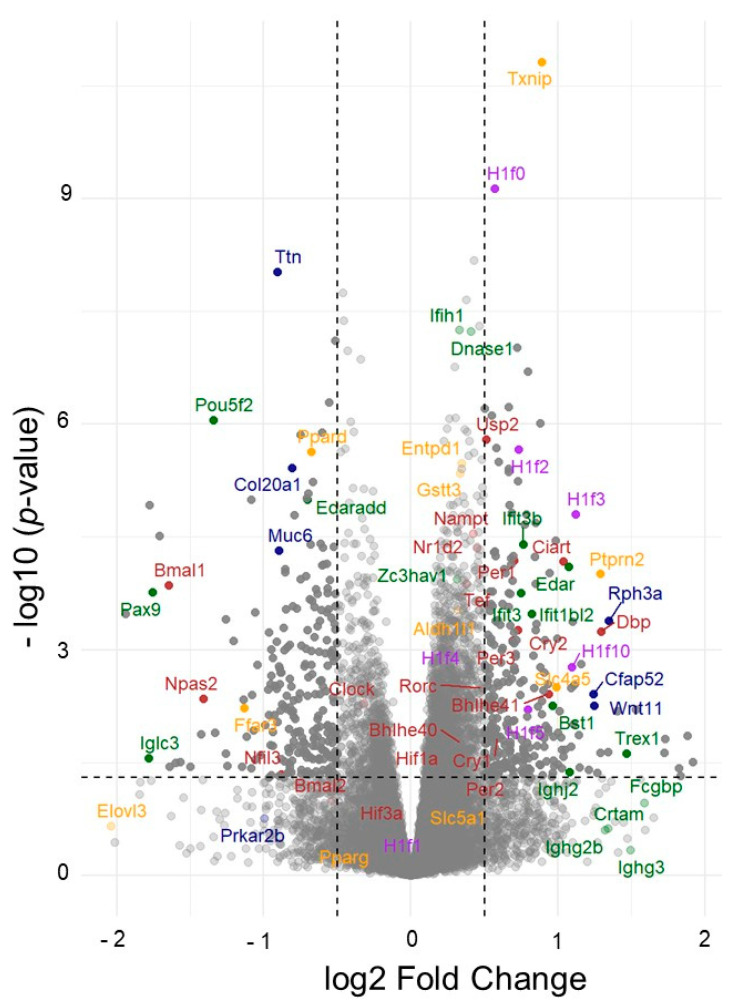
Volcano plots showing differential gene expression in diabetic mice treated with B12. The volcano plot displays the distribution of differentially expressed genes based on the log_2_ fold change (x-axis) and −log_10_(*p*-value) (y-axis). Vertical dashed lines indicate the fold change cutoff (±0.5), and the horizontal dashed line represents the significance threshold (*p* < 0.05). Genes that meet both thresholds are classified as significant and are plotted with higher opacity, while non-significant genes are shown with reduced opacity. Data points are color-coded according to functional categories: immune (dark green), histone (purple), circadian (red), metabolism and transport (orange), and structural (navy). Representative genes within each category are labeled. The top 20–30 genes for each category are shown in separate volcano plots; due to the multiple functions of *Txnip*, it is also categorized as antioxidant (Figure S4A–J).

**Figure 3:**
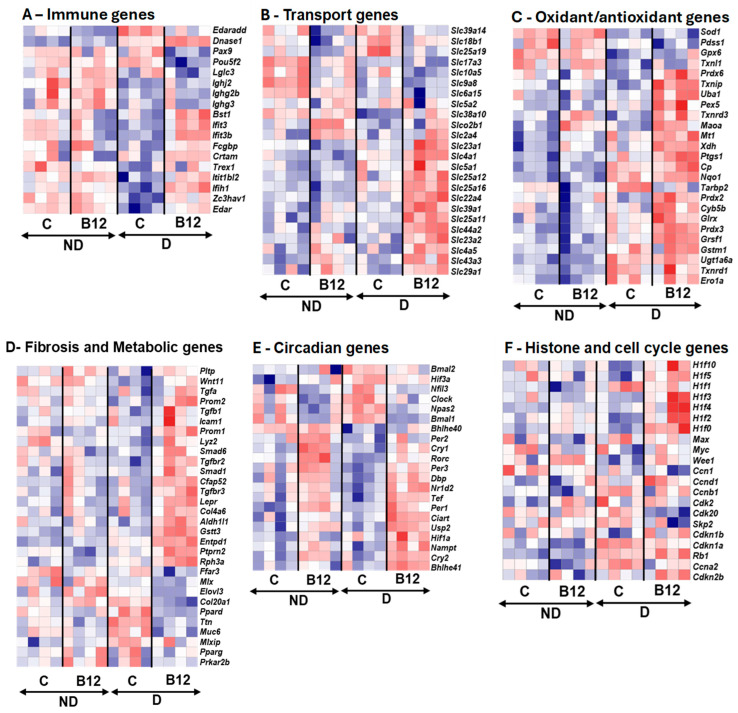
Vitamin B12 Modulates Gene Expression Across Multiple Pathways. Heat maps represent the expression of (**A**) immune genes; (**B**) transport genes; (**C**) oxidant/antioxidant genes; (**D**) fibrosis and metabolic genes; (**E**) circadian genes; and (**F**) histone and cell cycle genes in non-diabetic (ND) and diabetic (D) mice treated with Vitamin B12 (B12) or untreated controls (C). The color intensity represents normalized expression (log_2_ fold change in normalized counts), with red indicating upregulation and blue indicating downregulation. Selected genes were confirmed by RT-PCR, as presented in Figure S5.

The physiological and histological characterizations described above confirm that four months of age is the appropriate time point for investigating the protective effects of B12. At this stage, diabetic nephropathy in untreated diabetic mice has begun to develop at an early phase, and the global RNA expression profile is likely influenced both by inflammatory cell infiltration into the damaged kidneys and by secondary tissue responses. We therefore subjected four kidneys from each group to RNA seq analyses (*n* = 4 each). The differential gene expression analyses revealed widespread transcriptional differences between the kidneys of diabetic mice with and without B12 supplementation, as illustrated in a volcano plot (Figure 2). The top significantly upregulated and downregulated genes within each functional category, with their full names, and the effect of diabetes and B12 treatment, as well as the corresponding log2 fold-change values and padj values, are provided in Table S2. The mean normalized counts (±standard error) for each gene in each group, along with the effects of diabetes, B12 treatment, and their interactions, were assessed using a generalized linear model, as shown in Table S3. These representative gene groups were further analyzed alongside counts from non-diabetic kidneys and illustrated using heat maps (Figure 3). This analysis revealed that multiple genes previously associated with diabetic nephropathy are also responsive to B12. The significantly altered genes broadly belong to immune signaling (Figure 3A), transport (Figure 3B), redox pathways (Figure 3C), and fibrosis/metabolism (Figure 3D). In addition, genes involved in circadian rhythm regulation (Figure 3E) and linker histone H1 variants (Figure 3F) were among the top significantly regulated genes.

#### 3.2.1. Immune-Inflammatory Pathways

In B12-treated diabetic mice, *Edar*, a member of the tumor necrosis factor (TNF) receptor family that activates the downstream signaling pathway, including NF-kB, which is important for inflammation and tissue development, was upregulated. Its adaptor *Edaradd*, which is required to transmit signals from *Edar* to NF-κB, was downregulated, pointing to selective regulation in this signaling axis and NF-κB activation [17]. This dual pattern suggests that B12 preserved baseline EDAR function while limiting EDARADD-driven amplification of NF-κB–mediated inflammatory stress. Because *Edar* can also activate alternative pathways such as JNK/Erk signaling, B12 may bias downstream signaling toward protective, non-inflammatory outcomes [18]. Several interferon-stimulated genes (*Bst1*, *Ifit1bl2*, *Ifih1*) were also upregulated (Figure S4A), indicating that B12 restored interferon responsiveness, which is often impaired under hyperglycemia.

In addition, multiple relatively small-expression immunoglobulin genes that were suppressed in untreated diabetic kidneys (*Ighj2*, *Ighg1*, and *Iglc3*; counts are provided in Table S3.) were partially restored with B12, suggesting recovery of humoral defense mechanisms. While CD19^+^ B-cell infiltration was prominent in untreated diabetic kidneys, consistent with reports of their pathogenic role in DN, B12 treatment appeared to attenuate this pathological infiltration while enhancing T-cell-associated regulatory signatures. These results align with clinical evidence showing that B12 supplementation restores lymphocyte counts and enhances regulatory T-cell function in B12-deficient individuals [19]. Together, these findings support the immunomodulatory role of B12, as it alleviates renal inflammation by reducing pathogenic B-cell activity, promoting protective T-cell pathways (Figure S2A,B) and markedly lowering the elevated M1 macrophage population observed in diabetic kidneys (Figure S3).

#### 3.2.2. Solute Carrier Expression and Water Handling

Proximal tubular cells are the most metabolically active cells in the kidney [20], and genes encoding transporters that are essential for electrolyte, amino acid, and phosphate regulation (*Slc4a5*, *Slc22a4*, *Slc4a1*, and *Slc44a3*) were significantly upregulated in the B12-treated diabetic group, suggesting improved tubular homeostasis. Conversely, solute carriers such as *Slc10a5* and *Slc25a19* were suppressed, indicating selective downregulation of nutrient and ion transport. *Aqp6* expression was also upregulated, pointing to enhanced water reabsorption capacity (Figure S4B). Of note is that B12 strongly upregulated *Slc4a5*, which may explain the observed improvement in blood pressure regulation in the diabetic mice treated with B12.

#### 3.2.3. Redox Regulation

B12 is a SOD mimetic [3] and reduces oxidative stress on cells [1]. The current study did not show strong regulation of most of the antioxidant-related genes. Some of them, such as *Uba1*, *Pex5*, *Txnrd3*, and *Maoa*, were slightly upregulated, while *Sod1*, *Pdss1*, and *Gpx6* were suppressed in untreated diabetic kidneys but preserved with B12 (Table S2). Notably, *Txnip*, which responds to the glucose-inducible inhibitor of thioredoxin (Trx), was robustly induced with B12, which may appear maladaptive. This might seem harmful at first [21], but with B12, *Txnip* upregulation is likely be an adaptive response to manage oxidative stress and restore redox balance (Figure 3C). Together, these findings demonstrate that B12 strengthens renal antioxidant capacity and reduces diabetes-driven oxidative stress (Figure S4C).

#### 3.2.4. Metabolic and Structural Pathways

Fibrosis and extra cellular matrix (ECM)-associated genes such as *Col20a1*, *Muc6*, and *Ttn* were suppressed, while protective genes such as *Rph3a*, *Ptprn2*, *Aldoc*, and *Wnt11* were upregulated (Figure S4D–F). The heatmap analysis revealed the strong induction of fibrosis-associated and collagen-related genes in diabetic kidneys compared with controls. Multiple collagen isoforms, including *Col5a3*, *Col11a2*, *Col4a3*, *Col4a1*, *Col4a2*, *Col4a4*, *Col24a1*, *Col7a1*, *Col10a1*, and *Col20a1*, exhibited marked upregulation in the diabetic group, indicating enhanced extracellular matrix (ECM) remodeling and collagen deposition (Figures S1 and S6). These changes suggest that B12 reduces maladaptive structural remodeling while enhancing repair pathways. Though the overall expression level was low, the upregulation of *Cfap52* was particularly notable. This gene is best known for its role in ciliary function in sperm [22]; its induction in kidney tissue suggests broader restoration of cilia-associated pathways. Because cilia serve as sensory and polarity hubs in podocytes and tubular epithelia, *Cfap52* upregulation may stabilize epithelial organization and enhance renal function. Restoration of *Wnt11* further supports this interpretation, as *Wnt11* plays a crucial role in kidney polarity and epithelial repair [23]. Together, these changes demonstrate that B12 stabilizes multiple metabolic axes, including glucose handling, lipid metabolism, and mitochondrial function in the diabetic kidney.

#### 3.2.5. Vitamin B12 Reprograms Circadian Clock Networks and Chromatin Architecture in the Diabetic Kidney

B12 supplementation exerted pronounced effects on circadian regulation in the diabetic kidney (Figure 3E). Core clock activators including *Bmal1*, *Clock*, and *Npas2* were consistently suppressed, whereas negative feedback regulators (*Cry1-2*, *Per1-3*) and the NAD^+^-biosynthetic gene *Nampt* were strongly induced. This coordinated shift indicates that B12 actively reprograms circadian transcriptional feedback loops, rather than just protecting against metabolic stress. The induction of *Nampt*, a central regulator of NAD^+^ metabolism [24], highlights a direct mechanistic link between B12 supplementation, redox cofactor availability, and circadian timing. These findings reveal that B12 mediates circadian–metabolic networks under diabetic conditions, suggesting a broader systemic role for B12 in synchronizing renal metabolism with whole-body clock regulation (Figure S4G).

Multiple linker histone H1 variants exhibited dynamic responsiveness: *H1f0*, *H1f2*, and *H1f4* were upregulated (Figure 3F), while *H1f3*, *H1f5*, and *H1f10* showed an interaction effect (Table S2). This pattern suggests that B12 reinforces chromatin compaction and nucleosome stability, potentially enhancing transcriptional control under diabetic stress. In parallel, upregulation of *Wee1*, a key G2/M checkpoint kinase [25], together with downregulation of *Cdk20*, points to a coordinated role for B12 in restraining cell cycle progression and synchronizing it with circadian timing [26] (Figure 3F). Together, these changes indicate that B12 does not solely act as a metabolic cofactor but actively remodels nuclear architecture and checkpoint regulation in the diabetic kidney (Figure S4H). Furthermore, the transcriptomic evidence positions B12 as a multi-layered modulator of renal homeostasis, with effects spanning immune signaling, solute and water transport, oxidative balance, metabolic remodeling, circadian alignment, and chromatin regulation. Although histological changes remained modest at this early stage, RNA-seq revealed clear molecular reprogramming toward protective pathways.

### 3.3. Circadian Gene Expression of Proximal Tubular BU.MPT Cells

**Figure 4:**
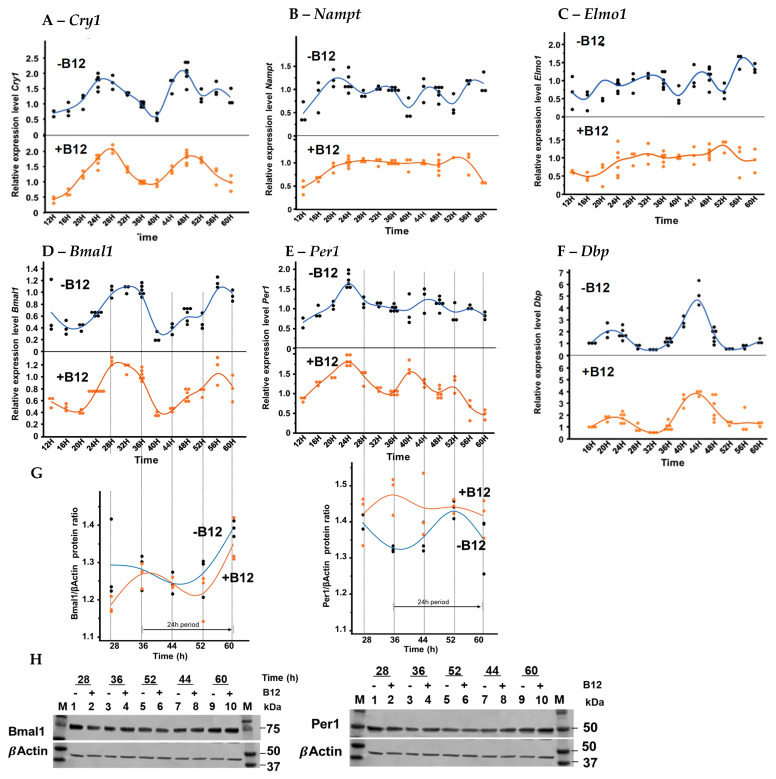
Effect of B12 on circadian gene rhythmicity in cultured BU.MPT cells. Cells were treated with B12 (+B12) or the vehicle (-B12) and gene expression was assessed by RT-qPCR across the circadian cycle. Control cells displayed strong oscillations in (**A**) *Cry1*, (**B**) *Nampt*, (**C**) *Elmo1*, (**D**) *Bmal1*, (**E**) *Per1*, and (**F**) *Dbp*, whereas B12 treatment altered the phase and amplitude of these rhythms. Data were normalized to *β-actin* expression, analyzed using the 2^−ΔΔCt^ method, and expressed as linear expression ratios, with the mean expression at 36H of the individual gene illustrated without B12 set to 1.0. Total protein extracts from BU.MPT cells synchronized with dexamethasone were collected at selected time points and analyzed by SDS–PAGE followed by (**H**) Western blotting for Bmal1 and Per1. (**G**) Graphs show the band intensity ratios normalized to β-actin. The experiment was performed once, and band quantification was carried out using Fiji ImageJ 1.54p (Wayne Rasband and contributors, National Institute of Health, Bethesda, MD, USA). Original figures can be found in Supplementary Materials.

B12 treatment significantly modified the oscillatory rhythms of several circadian and metabolic genes, including *Bmal1*, *Per1*, *Dbp*, *Cry1*, *Nampt*, and *Elmo1* in BU.MPT cells. Under control conditions (without B12), these genes displayed clear rhythmic patterns of relative expression with multiple distinct peaks across the time course (Figure 4). In the presence of B12 in the medium, the peaks for the core clock genes *Bmal1*, *Per1*, and *Dbp* were shifted forward by approximately 2–4 h, while the *Cry1* peak was delayed by about 2 h. Specifically, in the control medium, the two main peaks of *Bmal1* were observed at ~34 h and 58 h, respectively, whereas with B12, peaks were detected at ~30 h and ~56 h, with the second peak being lower in amplitude than the first. *Per1* normally showed peaks at 26 h, 46 h, and 56 h, but with B12 treatment, the peaks shifted to 24 h, 40 h, and 52 h, indicating a consistent shift forward. *Dbp* showed only a slight phase shift: its early peak at ~22 h remained unchanged, but the later peak shifted from 44 h (control) to 42 h (B12). By contrast, *Cry1* displayed three peaks (two of which were major), under control conditions (24 h, 47 h, and 56 h), but with B12 treatment, only two peaks were observed, at ~28 h and ~51 h. This reflects a delay with B12 treatment, with no significant difference in the amplitude of these two genes.

In contrast, the metabolic regulators *Nampt* and *Elmo1* lost their normal oscillatory behavior under B12 treatment. In the control medium, both genes showed four clear peaks across the monitoring period. With B12, however, their oscillations flattened, remaining at a consistent level, particularly from 20 h to 52 h, suggesting a loss of rhythmic oscillation and a shift toward sustained expression (Figure 4).

Protein levels in the BU.MPT cells exhibited clear time-dependent oscillations of core clock proteins. Bmal1 protein showed a rhythmic pattern in both the control and B12-treated groups, with a shared trough around 44 h and rising levels toward 60 h. However, B12 treatment reduced the overall Bmal1 amplitude, resulting in lower expression at several time points compared with controls. These findings indicate that while phase timing remained largely preserved, B12 attenuates the magnitude of Bmal1 rhythmic expression, suggesting a modulatory effect on the positive limb of the clock. Per1, a negative-arm clock component, also displayed rhythmic oscillation with a trough near 36 h and a peak around 52 h in control cells, consistent with its expected phase delay relative to Bmal1. In contrast to Bmal1, B12-treated cells showed higher baseline Per1 levels and a smoother, more stable oscillatory pattern. This deviation in Bmal1 dampening versus Per1 elevation suggests that B12 differentially modulates positive and negative clock components in renal epithelial cells. Together, these data confirm that BU.MPT cells maintain an intrinsic circadian rhythm after synchronization and raise the possibility that B12 influences circadian protein dynamics through metabolic or epigenetic pathways, though additional studies are required to define the underlying mechanisms (Figure 4G,H).

## 4. Discussion

In this study, we aimed to determine the basis of the reno-protective effects of B12 in early DN, linking pathological observations with transcriptomic analysis. The genes significantly regulated by B12 include those (1) showing immuno-modulatory functions restoring interferon responsiveness and limiting CD19-positive B-cell infiltration while enhancing regulatory T-cell function; (2) involved in the maintenance of tubular cell homeostasis including cilial repair, solute carrier expression, and mitochondrial metabolic functions; (3) showing enhanced redox regulation and antioxidant defense capacity; (4) involved in the promotion of tissue repair and fibrosis; and (5) involved in the regulation of circadian rhythm and cell cycle progression. These B12-regulated genes include *Col20a1*, *Txnip*, *Ptprn2*, and *Ttn*, which have also been implicated in genome-wide association studies in humans with DN [27,28] or discovered by the use of animal models [27,28], and which are all worthy of focused analyses and evaluations in the future. Within the scope of our current work, we will focus the following discussion on potential roles of the circadian rhythm control of B12-affected genes in diabetic complications in kidneys.

Previous studies demonstrate that B12 supplementation can broadly affect DNA methylation patterns in human cells and in vivo, likely via one-carbon metabolism and SAM production [29]. Moreover, there is growing evidence that core clock genes are regulated epigenetically via promoter and histone modifications [30], which could provide a mechanistic link for how B12 alters circadian gene expression in peripheral tissues. However, no studies have to date examined the effects of B12 on these genes or on other B12-regulated genes which were found in our study. In addition, the B12-regulated genes described and the precise mechanisms underlying their kidney-protective effects remain to be elucidated.

The circadian clock is an intrinsic timing system that regulates physiological and behavioral functions in alignment with the light–dark cycle through transcriptional–translational feedback loops [31]. We did not anticipate a priori that Vitamin B12 would affect the expression of circadian rhythm-controlling genes in diabetic mice to a measurable extent, though the accumulating literature suggests its potential role. In the present study, B12 modulated several core clock genes (*Bmal1*, *Cry1*, *Cry2*, *Per1*, *Per3*), while other genes were primarily affected by diabetes (*Bhlhe40*, *Hif3a*, and *Nr1d1*). Additionally, a subset showed combined effects (*Clock*, *Per2*, *Bhlhe41*, and *Hif1a*). The publicly available data bank, Circa (http://circadb.hogeneschlab.org, accessed on 23 November 2025), lists daily oscillations in expression in multiple B12-responsive genes involved in immunity, antioxidant defense, and fibrosis pathways in peripheral tissues, including the kidney (Table S3) [32,33]. Thus, influence of B12 on circadian regulation likely contributed to the improvement of physiological parameters during the early stages of diabetic complications in our experiments.

Notably, circadian regulation is tightly interconnected with blood pressure (BP) control and kidney disease. Mice lacking *Cry1/2* show elevated daytime BP and worsened renal injury, whereas kidney-specific *Bmal1* disruption alters BP rhythms [34,35]. The fact that our samples (collected at ZT7–ZT9) showed lower clock activators and higher *Cry1/2* repressor expression may therefore have contributed to the SBP effects we observed with B12, even if these were not directly causal. In addition, contrary to reports that glutamine and methionine upregulate *Bmal1* in adipose tissue [32], B12 downregulated *Bmal1* in the diabetic kidney in our study, highlighting the tissue-specific regulation of circadian genes. In addition, recent works support a connection between solute carrier (SLC) gene expression and circadian clock regulation. For example, *Slc22a2*, a renal organic cation transporter, exhibits rhythmic expression controlled by the core clock protein CLOCK via PPARα-mediated signaling [33]. Similarly, *Slc5a1* (*Sglt1*), a sodium-glucose co-transporter, has been shown to follow a circadian pattern of expression in intestinal and renal tissues, influenced by transcription factors such as *Hnf1* and *Per1* [36]. In our study, we observed modulation in response to the B12 treatment of several SLC genes, including *Slc5a1* and some genes in the SLC22 family. While the precise mechanisms remain to be clarified, the coordinated changes in clock-controlling genes and SLC expression suggest that B12 influences metabolic transport through circadian pathways.

*Nampt* upregulation in B12-treated kidneys links circadian control and cell metabolism by enhancing the NAD^+^–Sirt1 feedback loop that modulates Clock:Bmal1 activity. *Nampt* encodes the rate-limiting enzyme of the NAD^+^ salvage pathway, a critical regulator of cellular energy metabolism [24,37]. In this context, the observed profile of low *Bmal1*/*Npas2*/*Clock* and high *Cry1-2* suggests that B12 enhances Nampt–NAD^+^–Sirt1 signaling, rebalancing circadian outputs in the kidney. The upregulation of *Hif1a* alongside circadian changes highlights a broader link between B12 and metabolic adaptation under stress. *Hif1a* is a master regulator of hypoxia responses and has been implicated in obesity and metabolic dysfunction [38,39]. Thus, B12 may reset peripheral clocks and partially normalize hypoxia-related signaling, providing a protective mechanism in metabolic disease.

An additional noteworthy finding in our study was the regulation of H1 linker histones by B12 treatment in diabetic mice. Linker histones occupy the nucleosome entry–exit regions of DNA, facilitate higher-order chromatin folding, and suppress aberrant transcriptional activity [40,41,42]. Our results suggest that B12 modulates the expression of histone linker genes and thereby influences epigenetic regulation, potentially reshaping chromatin structure and altering the transcriptional activity of genes related to metabolism, circadian rhythms, and renal function. Hyperglycemia, advanced glycation end-products (AGEs), and ROS remodel the chromatin structure, in part by shifting the activity of histone-modifying enzymes. Glycation can directly damage H1 proteins and weaken H1–chromatin interactions, leading to chromatin relaxation and the inappropriate activation of pro-inflammatory genes [43,44]. Such stress and dedifferentiation states are typically accompanied by reduced H1.0 levels, consistent with our observation in untreated diabetic mice.

Our experiments with a BU.MPT cell line demonstrated that B12 influences the oscillations of circadian genes, despite the patterns of mRNA levels and protein concentration being complex, providing an indication of B12’s potential regulatory role in circadian dynamics. B12 shifted oscillator timing in a non-uniform manner, and this deviation between positive- and negative-limb genes suggests that B12 alters the internal balance of the feedback loops, temporarily reshaping the expression of downstream clock-controlled genes. Several mechanisms could underlie this effect: The first is methylation control—B12 is a cofactor in one-carbon metabolism, potentially modifying DNA and histone methylation at clock gene promoters [45]. The second is NAD^+^ availability—by influencing *Nampt* and the NAD^+^ salvage pathway, B12 may alter the Sirt1-mediated deacetylation of Bmal1/Clock [46]. The third is protein synthesis and stability- B12 may affects protein synthesis/stability/ nuclear translocation. Further studies required. Our results are also consistent with prior evidence that methyl donor availability alters clock phase and amplitude. In hepatocytes, methylation-dependent modification of Bmal1/Clock binding sites shifted circadian oscillations [47]. In humans, B12 supplementation advanced melatonin and core body temperature rhythms, further supporting an entrainment role for B12 in peripheral clocks [48]. Finally, B12’s effects as a strong antioxidant cannot be ignored, since N-acetyl cysteine administration has been shown to delay the early onset of aging in mice lacking Bmal1 and extend their life span by reducing their chronic oxidative stress (Kondratov RV et al., Aging, vol1 No12 979).

While our current study provides novel insights into the effects of B12 on circadian clock gene expression in male *Elmo1*-overexpressing diabetic and non-diabetic mice, several limitations should be acknowledged. We have studied mice at only one time point, at ZT 7-9. Because circadian gene expression is inherently dynamic, multiple time points must be analyzed to fully capture the oscillatory patterns and the complete influence of B12 on circadian regulation. In addition, only male mice were studied due to the limitation of diabetic mouse models, which limits the generalizability of the findings. Sex-specific differences in metabolism, circadian rhythms, and the response to B12 may exist, and future studies should include female mice to evaluate these potential effects. The potential effect of altered clock gene expression may be associated with epigenetic modifications induced by Vitamin B12. Further studies are required to confirm this mechanism. The mouse model we used overexpresses the *Elmo1* gene and exhibits accelerated progression of DN. *Elmo1* is an established DN-linked locus, and further studies are necessary to elucidate how this locus interacts with B12 and with circadian rhythm.

## 5. Conclusions

Vitamin B12 exhibits protective effects against early diabetic kidney injury by modulating genes involved in redox balance, inflammation and immunity, solute transport, and structural integrity. The increased transcription of linker histone H1 variants suggests a potential role for enhanced chromatin stability and altered cell cycle progression. Together with our RNA-seq findings and BU.MPT cell oscillation data, these results point to a previously unrecognized epigenetic and circadian mechanism through which Vitamin B12 mitigates early diabetes-induced renal injury. The observed changes in cellular components involved in the renal response to diabetes further support a link between circadian gene regulation and H1 histone variants. Moreover, existing circadian datasets indicate that a substantial proportion of B12-responsive genes associated with DN are under circadian control. Collectively, these findings highlight the importance of circadian and epigenetic regulation in the protective actions of Vitamin B12 during early diabetic kidney injury.

## Data Availability

RNA sequencing data have been deposited in NCBI Gene Expression Omnibus (accession #: GSE306999).

## Supplementary Materials

The following supporting information can be downloaded at https://www.mdpi.com/article/10.3390/biom15121689/s1: Figure S1: Masson’s Trichrome staining highlights fibrosis and hematoxylin and eosin (H&E) staining of kidney sections to assess tissue morphology and structural changes. Figure S2: B-cell and T-cell distribution in diabetic kidneys with/without Vitamin B12 treatment; Figure S3: Double immunostaining of kidney sections for F4/80 (macrophage marker) and iNOS (M1 macrophage marker) for diabetic groups. Figure S4: Volcano plots of differentially expressed genes in B12-treated diabetic animals, categorized into functional groups; Figure S5: RT-PCR validation of selected genes. Figure S6: Vitamin B12 modulates gene expression across kidney fibrosis. Table S1: Real-time PCR primers used; Table S2: Top significantly altered genes within each functional category showing effects of diabetes and Vitamin B12 treatment [18,21,22,23,24,25,26,32,33,36,37,40,41,42,49,50,51,52]; Table S3: Mean normalized counts (±SE) for each gene and the effects of diabetes, Vitamin B12 treatment, and their interaction, as assessed by a generalized linear model.

## Abbreviations

The following abbreviations are used in this manuscript:

B12	Vitamin B12
SOD	Superoxide dismutase
ROS	Reactive oxygen species
*Elmo1*	Engulfment and Cell Motility 1
SNPs	Single nucleotide polymorphisms
GSH	Glutathione
TGFβ1	Transforming growth factor β1
SBP	Systolic blood pressure
GEO	NCBI Gene Expression Omnibus
qRT-PCR	Quantitative Reverse-transcription Polymerase Chain Reaction
PAS	Periodic Acid–Schiff
TNF	Tumor necrosis factor
ECM	*extra cellular matrix*
AGEs	Advanced glycation end-products
BP	Blood pressure
SLC	Solute carrier
