# Supplementary material for: Vitamin B12 Protects Against Early Diabetic Kidney Injury and Alters Clock Gene Expression in Mice
Source: Biomolecules. 2025 Dec 3;15(12):1689. doi: 10.3390/biom15121689 (PMC12731228; doi:10.3390/biom15121689)
Supplement: Supplementary file 1 [file biomolecules-15-01689-s001.zip › revised supplimentary figures S1_S2_S3_S4_S5_S6_Table S1_Table S2.pdf]

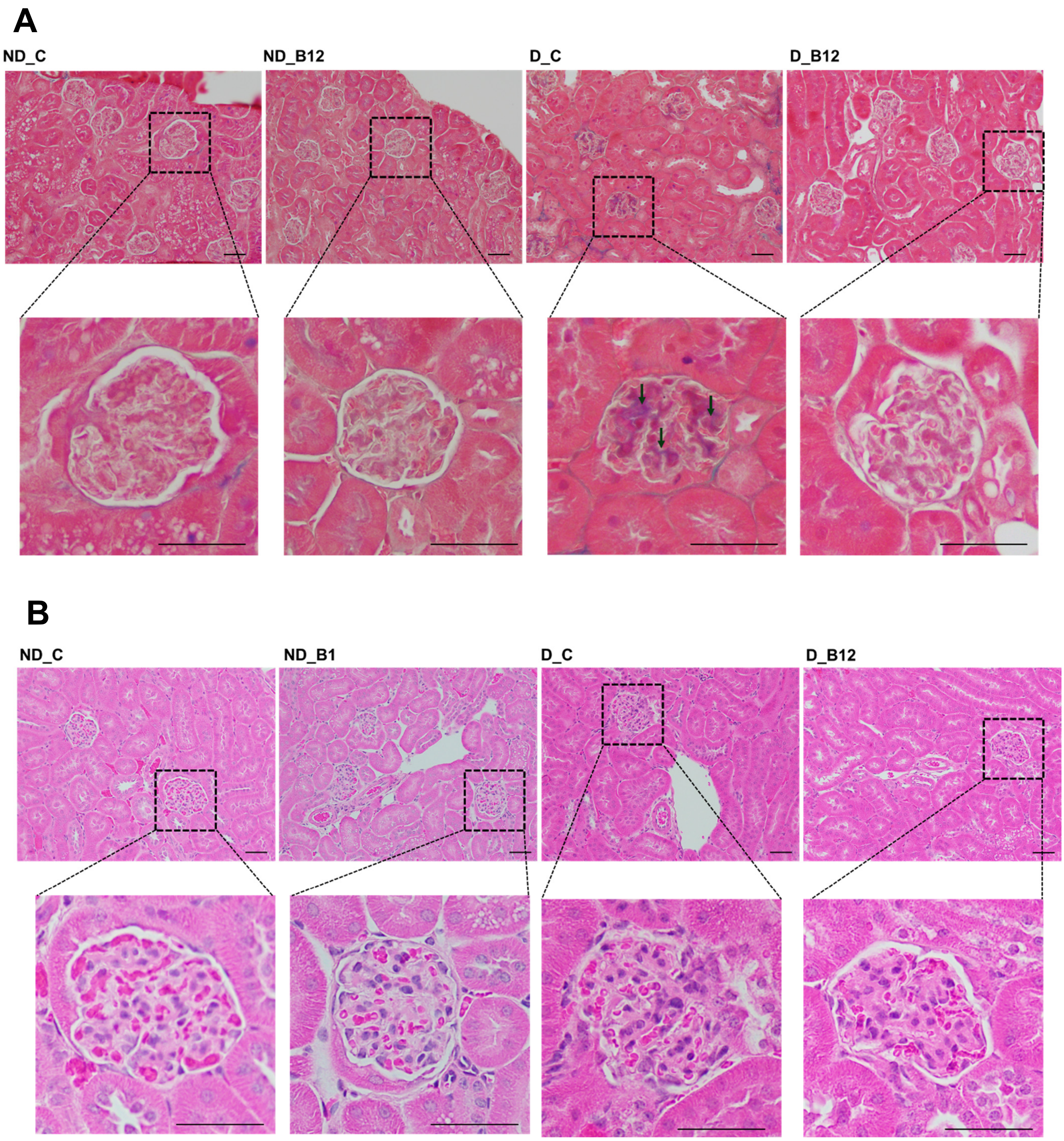

Figure S1: (A) Masson's Trichrome staining highlights fibrosis (green arrows) and High magnification images of the glomerulus in trichome staining. (**B**) Hematoxylin and eosin (H&E) staining of kidney sections to assess tissue morphology and structural changes. Groups: non-diabetic (ND), diabetic (D), control (C), vitamin B12–treated (B12). Scale bars: 50 µm.

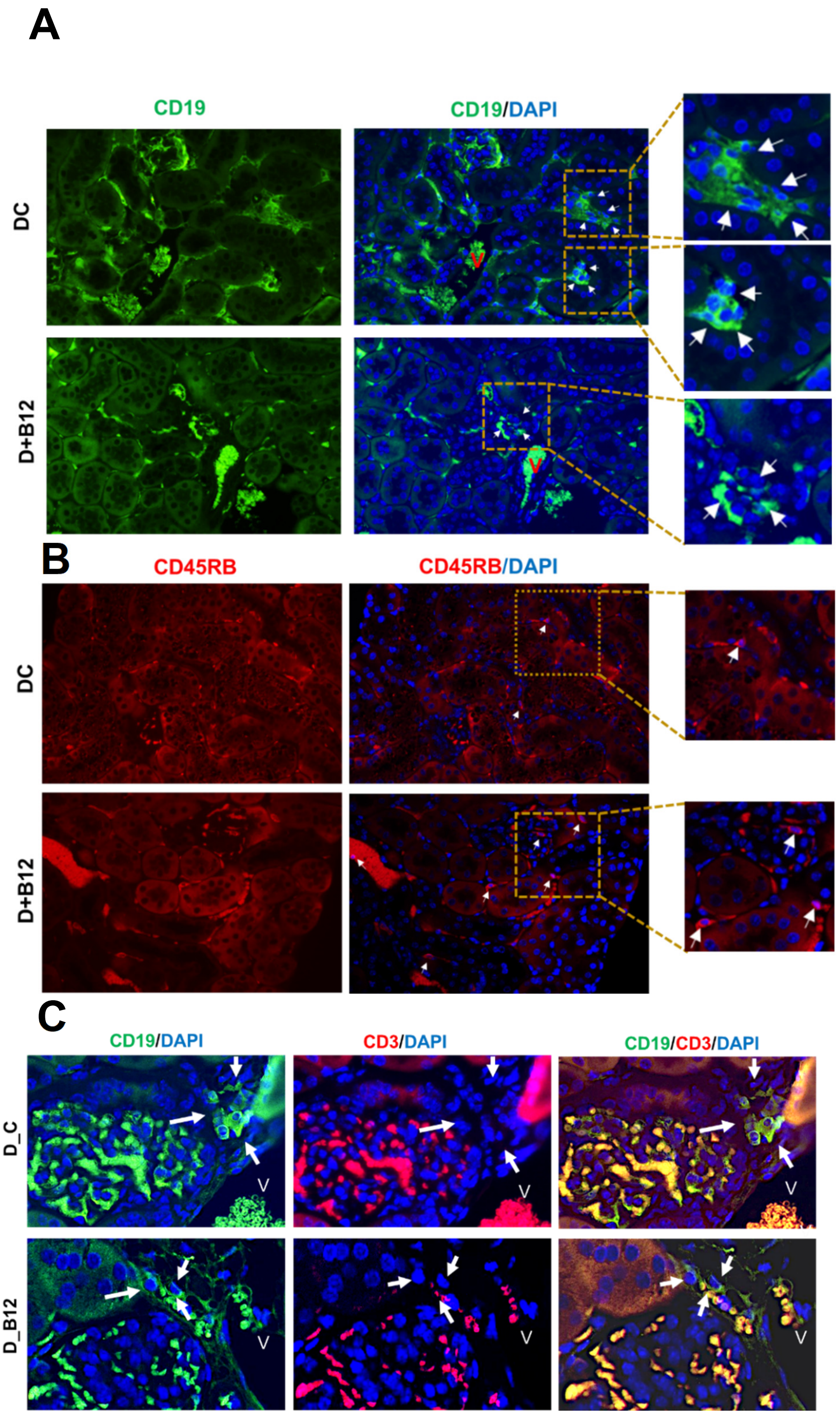

Figure S2: B cell and T cell distribution in diabetic kidneys with and without Vitamin B12 treatment. Immunostaining of kidney sections was performed to assess the infiltration of (A) B cell, (B) T cell. Double staining (C) identified B cells and T cells in the clusters. We observed an increased number of B cell–positive signals in diabetic kidneys compared with Vitamin B12–treated diabetic kidneys, indicating elevated B cell infiltration in the untreated diabetic kidneys. In contrast, T cell staining was comparatively higher in Vitamin B12–treated diabetic kidneys, whereas untreated diabetic kidneys exhibited lower T cell presence. Only B cells were identified in the clusters in both treated and non-treated mice. Magnification 20X. Arrow: Positive B cells clusters (CD19); Naive T cells (CD45RB); DAPI: nuclei; V: Blood vessel

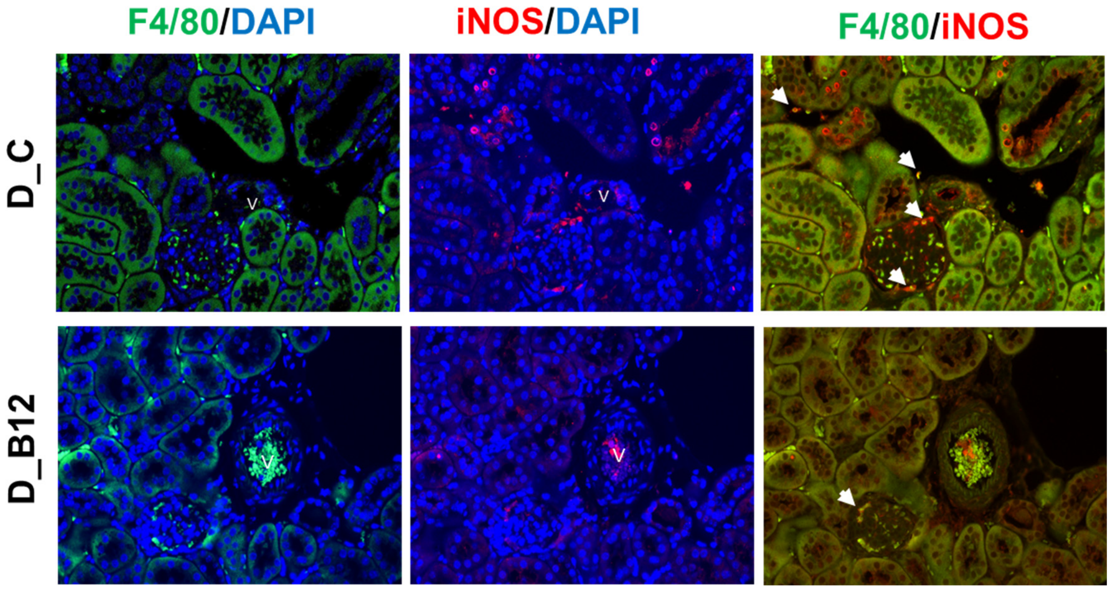

Figure S3: Double immunostaining of kidney sections for F4/80 (macrophage marker) and iNOS (M1 macrophage marker) in control (C), diabetic (D), and Vitamin B12-treated diabetic (D_B12) mice. White arrows indicate iNOS-positive (activated, M1) macrophages. Magnification 20X. V: Blood vessels.

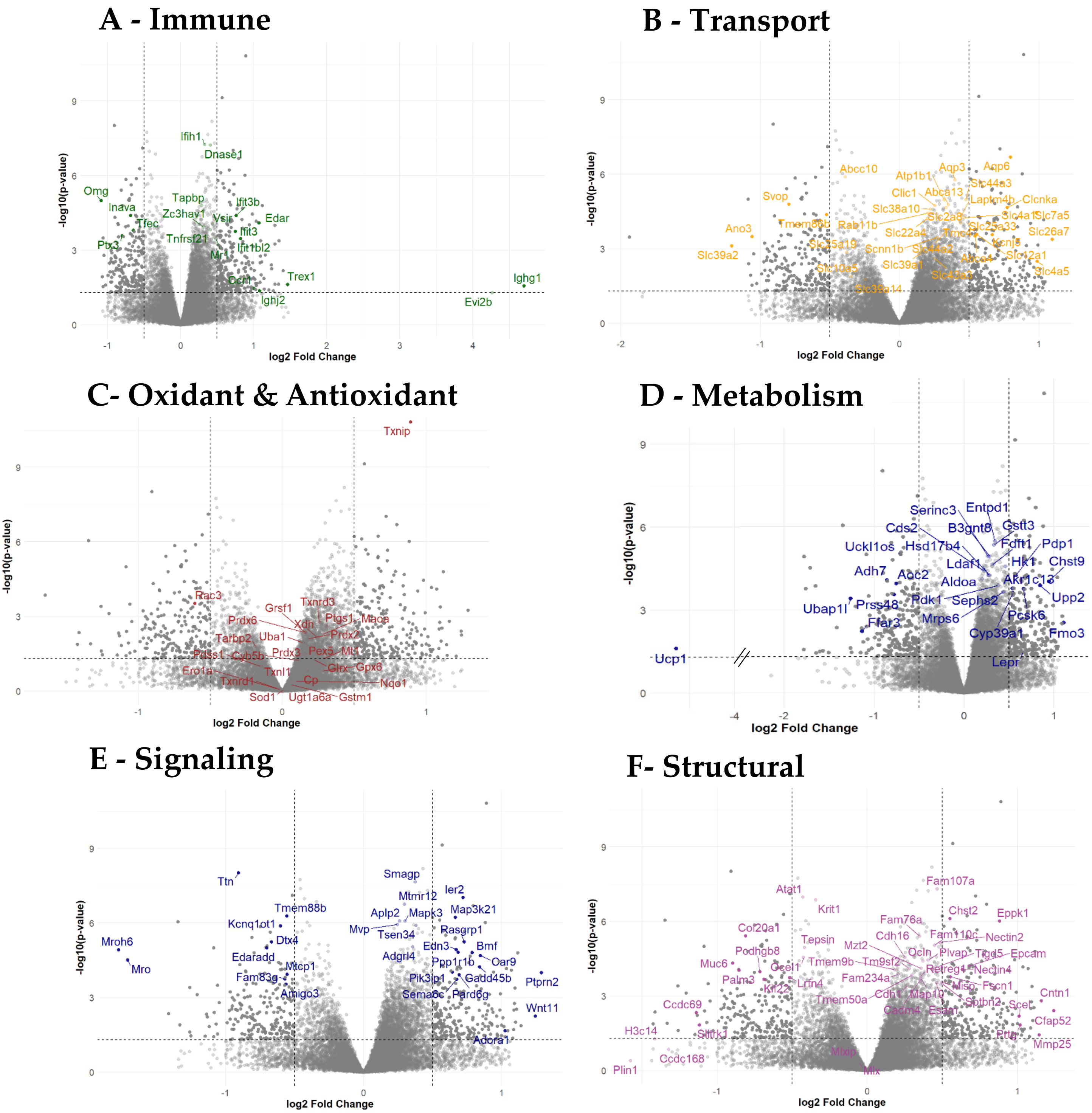
A - Immune
B - Transport
C- Oxidant & Antioxidant
D - Metabolism
E - Signaling
F- Structural
-log10(p-value)
log2 Fold Change
Ifih1
Dnase1
Omg
Tapbp
Ifit3b
Inava
Zc3hav1
Vsir
Edar
Tfec
Ifit3
Tnfrsf21
Ifit1bl2
Ptx3
Mr1
Trex1
Ighg1
Ccl1
Ighj2
Evi2b
Abcc10
Aqp3
Aqp6
Atp1b1
Slc44a3
Svop
Clic1
Abca13
Lapim4b
Clcnka
Slc38a10
Slc2a8
Slc4a1
Slc7a5
Ano3
Tmem50b
Rab11b
Slc25a33
Slc26a7
Slc22a4
Slc35a19
Scnn1b
Kcnj8
Slc39a2
Slc12a1
Slc39a1
Slc4a5
Txnip
Rac3
Grsf1
Txnrd3
Prdx6
Ptgs1
Maoa
Xdh
Tarbp2
Uba1
Prdx2
Prss1
Cyb5b
Prdx3
Pex5
Mt1
Ero1a
Txnl1
Glrx
Gpx8
Txnrd1
Cp
Nqo1
Sod1
Ugt1a6a
Gstm1
Serinc3
Entpd1
Cds2
B3gnt8
Gstt3
Uckl1os
Hsd17b4
Fdft1
Pdp1
Ldaf1
Hk1
Chst9
Adh7
Aoc2
Aldoa
Akr1c13
Ubap1l
Prss48
Pdk1
Sephs2
Upp2
Ffar3
Mrps6
Pcsk6
Cyp39a1
Fmo3
Ucp1
Lepr
Smagp
Ttn
Ier2
Mtmr12
Tmem88b
Aplp2
Mapk3
Map3k21
Kcnq1ot1
Mvp
Rasgrp1
Tsen34
Mroh6
Dtx4
Edn3
Bmf
Edaradd
Adgrl4
Ppp1r1b
Oar9
Mro
Mtcp1
Fam83g
Pik3ip1
Gadd45b
Ptprn2
Amigo3
Sema6c
Pard6g
Wnt11
Adora1
Fam107a
Atat1
Krit1
Chst2
Eppk1
Fam76a
Col20a1
Cdh16
Fam110c
Nectin2
Tepsin
Mzt2
Podhgb8
Ocln
Plvap
Tgd5
Epcam
Muc6
Tmem9b
Tm9sf2
Gcel1
Refreg4
Nectin4
Palm3
Fam234a
Kif22
Lrfn4
Misp
Fscn1
Cntn1
Cdh1
Map10
Ccdc69
Tmem50a
Sptbn2
Scel
Cadm4
Esam
Cfap52
H3c14
Slitrk1
Prtg
Mmp25
Mixip
Ccdc168
Plin1
Mix

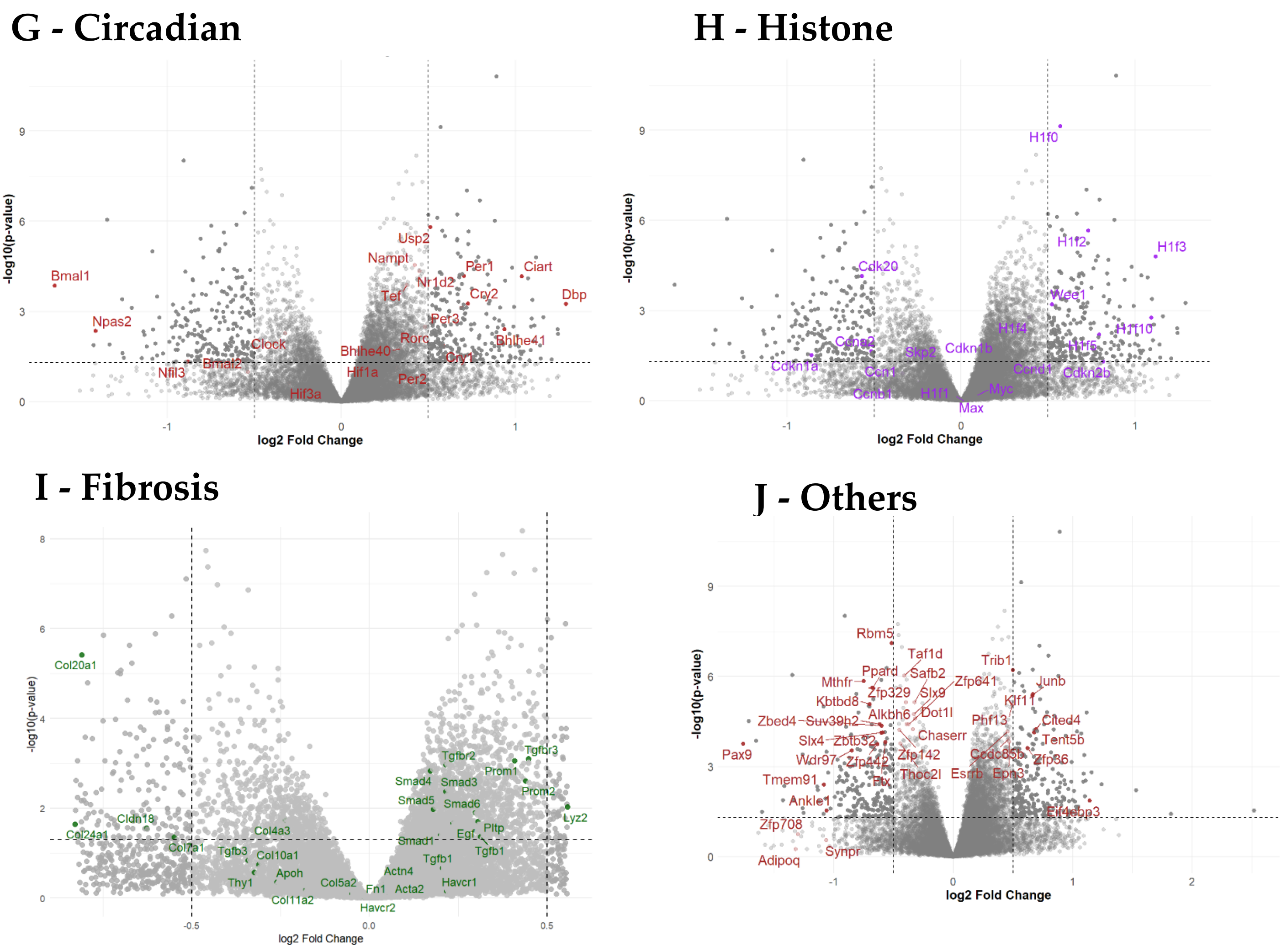

Figure S4: *Volcano plots of differentially expressed genes in B12-treated diabetic animals, categorized into functional groups*. Each panel represents genes belonging to a selected category: (**A**) Immune, (**B**) Transport, (**C**) Oxidant & Antioxidant, (**D**) Metabolism, (**E**) Signaling, (**F**) Structural, (**G**) Circadian, (**H**) Histone, (**I**) Fibrosis and (**J**) Others. The x-axis shows the $\log_2$ fold change, and the y-axis shows the $-\log_{10}(p\text{-value})$. Dotted vertical lines represent the ±0.5 $\log_2$ fold change threshold, and the dotted horizontal line indicates the p-value cutoff of 0.05. Genes belonging to the highlighted category are labeled, while other genes are shown in grey, red (upregulated), or blue (downregulated).

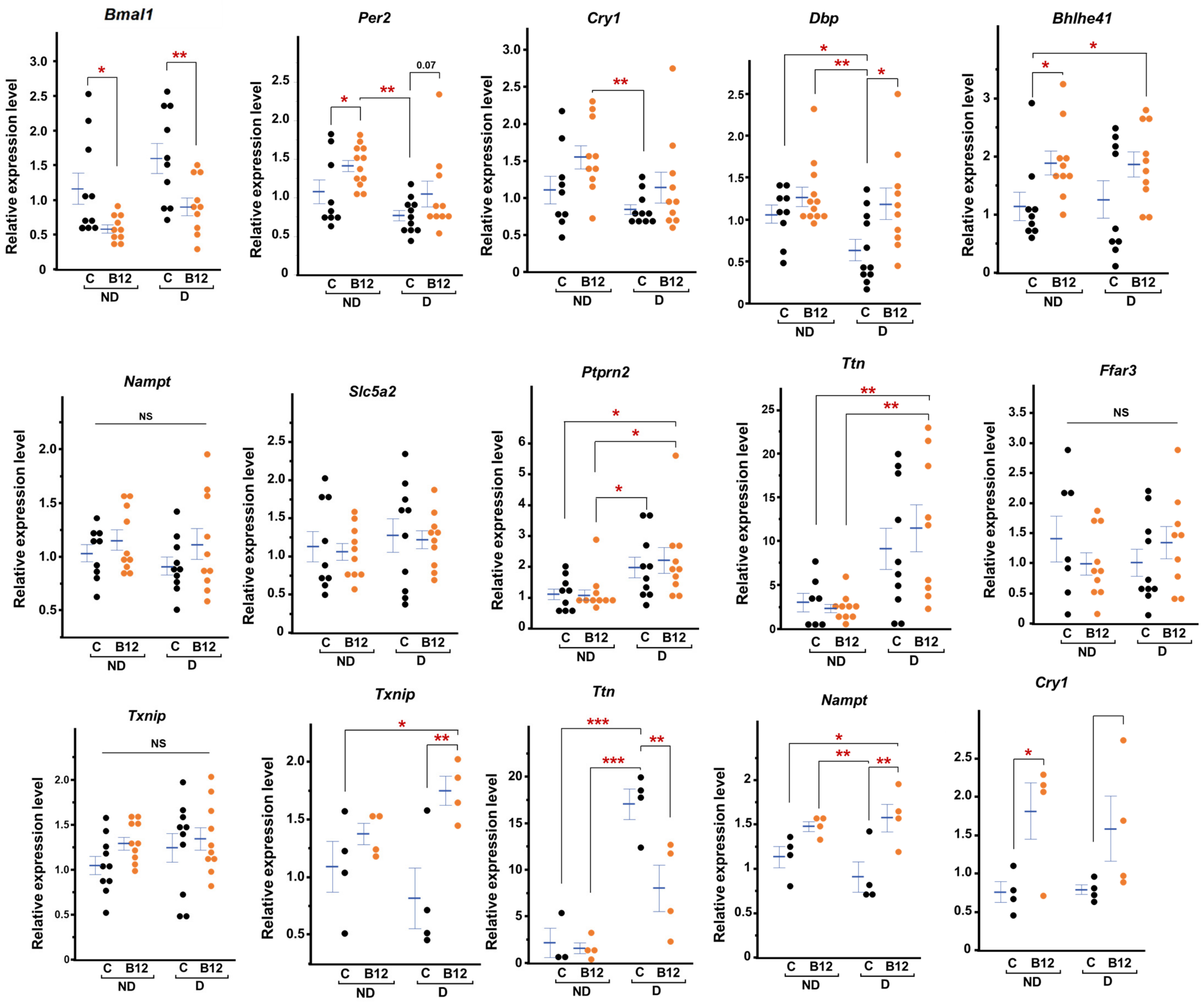

Figure S5: *RT-PCR validation of selected genes*. Experimental groups: non-diabetic (ND) and diabetic (D) mice treated with vitamin B12 (B12) or control (C). Data represent n = 9–10 mice per group. *$P < 0.05$, **$P < 0.001$, ***$P < 0.0001$; NS, not significant. (Note: Additional figures for *Txnip*, *Ttn*, *Nampt*, and *Cry1* with only the animals used for RNA-seq analysis (n = 4)).

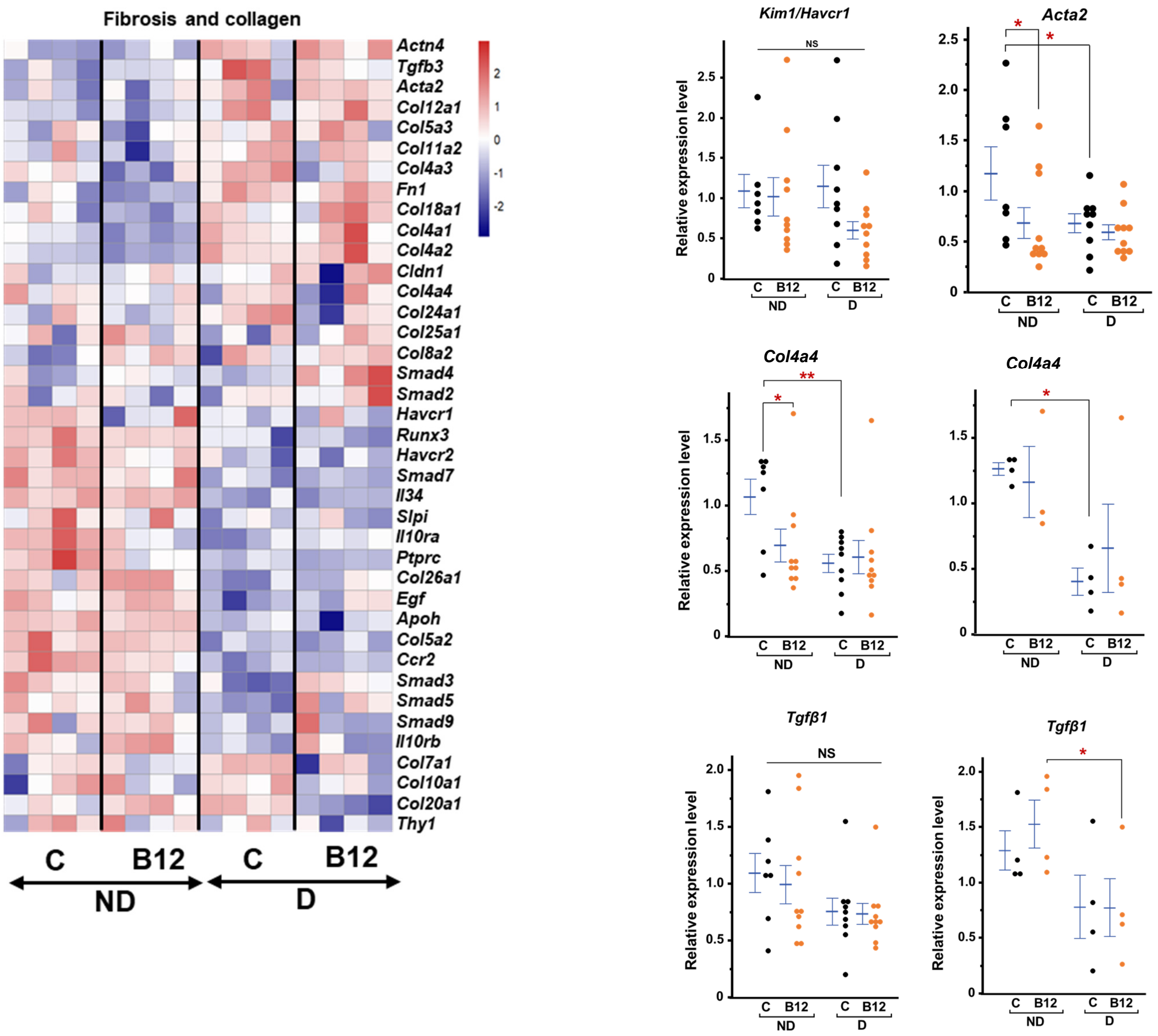

Figure S6: ***Vitamin B12 Modulates Gene Expression Across Kidney Fibrosis****.* Color intensity represents normalized expression (log_2 fold change of normalized counts), with red indicating upregulation and blue indicating downregulation. Groups: non-diabetic (ND), diabetic (D), control (C), vitamin B12–treated (B12). *$P < 0.05$, **$P < 0.001$; NS, not significant. (Note: Additional figures for *Col4a4*, and *Tgfβ1* with only the animals used for RNA-seq analysis (n = 4)).

Table S1. Real-Time PCR primers used

Gene symbol	Primers and Probes	Sequence	Assay ID
Actb	Forward primer	**5'**-AAGAGCTATGAGCTGGA-**3'**	
	Reverse Primer	**5'**-ACGGATGTCAACGTCACACT-**3'**	
	Probe	**5'-FAM**-CACTATTGGCAACGAGCGGTTCCG-**Tamra-3'**	
Arnt1(Bmal1)	Forward primer	**5'**-CAGCCCGCTGAACATCACAA-**3'**	
	Reverse Primer	**5'**-GTCCCTCCATTTAGAATCTTC-**3'**	
	Probe	**5'-FAM-**ACGCCTCCCCCTGATGCCTCTTC-**Tamra-3'**	
Dbp	Forward primer	**5'**-AAAGTCCAGGTGCCTGAGGA-**3'**	
	Reverse Primer	**5'**-CCTCTTGGCTGCTTCATTGT-**3'**	
	Probe	**5'-FAM-**ACCTCCGGCTCCAGTACTTCTCATC-**Tamra-3'**	
Bhlhe41	Forward primer	**5'**-GCAGCATCAGAAGATAATTGC-**3'**	
	Reverse Primer	**5'**-GCATCCAAGTCGGCCTGGA-**3'**	
	Probe	**5'-FAM-**CGATTTCAGAGAGCGCTCCCCATTC-**Tamra-3'**	
Slc5a2	Forward primer	**5'**-AGGAGCTGCTGCTAGTTGGA-**3'**	
	Reverse Primer	**5'**-CTGCACCACTGGCAGCCAA-**3'**	
	Probe	**5'-FAM-**ACGGACACCGCCACGATGAATACCA-**Tamra-3'**	
Ttn	Forward primer	**5'**-CTTTGAGGCTCACGTTAGTG-**3'**	
	Reverse Primer	**5'**-GTGGAAGTTGAAATCACCTGG-**3'**	
	Probe	**5'-FAM-**TCCCCAGTTCCTGAGGTGAGCTGG-**Tamra-3'**	
Cry1	Forward primer	**5'**-ATGGAGGGCTCATGGGCTAT-**3'**	
	Reverse Primer	**5'**-CCACTTCCTTGAGAGCAATTTC-**3'**	
	Probe	**5'-FAM-**CTGGAGAGAACGTCCCGAGCTGTA-**Tamra-3'**	
Havcr1	Forward primer	**5′**-CAAGTTAAACCAGAGATTCCCAC-**3′**	
	Reverse Primer	**5′**-TGAAATAGCCGTGGGTCTTC-**3′**	
	Probe	**5'-FAM-**TCCTCCAAGAAGACCCACAACTACAAG-**Tamra-3'**	
Acta2	Forward primer	**5′**-CGCTGTCAGGAACCCTGAGA-**3′**	
	Reverse Primer	**5′**-CGAAGCCGGCCTTACAGAG-**3′**	
	Probe	**5'-FAM-**CAGCACAGCCCTGGTGTGCGAC-**Tamra-3'**	

Col4a4	Forward primer	**5′-**GGCTATTCCTTCGTGATGCA**-3′**	
	Reverse Primer	**5′-**CTAAACTCTTCCAGACAGGAC**-3′**	
	Probe	**5'-FAM-**AGGTTCCGGCCAAGCCCTCGC**-Tamra-3'**	
Tgfβ1	Forward primer	**5′-**TGCTTCAGCTCCACAGAGAA**-3′**	
	Reverse Primer	**5′-**GTGGATCCACTTCCAACCCA**-3′**	
	Probe	**5'-FAM-**CCTTCCTAAAGTCAATGTACAGCTGCCG- **Tamra-3'**	
Per2			Mm00478099_m1
Nampt			Mm00451938_m1
Ptprn2			Mm01229147_m1
Ffar3			Mm02621638_s1

Table S2. Top significantly altered genes within each functional category showing effects of diabetes and vitamin B12 treatment

Gene	log2FC	*Padj*	Full gene name	Role in diabetic nephropathy	Reference	Effect
Vsir	0.44	<0.01	V-set immunoregulatory receptor (VISTA)	Immune checkpoint regulator — modulates T cell activation and renal inflammation.		D,B12, I
Edaradd	-0.7	<0.001	EDAR-associated death domain	Modulates NF-κB/apoptosis signalling; may influence renal cell survival/inflammation	[18]	D, B12, I
Ifih1	0.33	<0.0001	interferon induced with helicase C domain 1 (MDA5)	Innate immune sensor; may modify renal immune responses in DN.		B12, I
Bst1	0.97	0.07	bone marrow stromal cell antigen 1 (CD157)	NADase / immune regulator; possible role in inflammation and renal immune modulation.		B12, I
Ifit1bl2	0.82	<0.05	interferon induced protein 1B-like 2	Potential role in DN inflammation signatures.		D, B12
Edar	1.07	<0.01	ectodysplasin A receptor	TNF-family receptor; immune signaling and apoptosis modulation.	[18]	D, B12, I
Ptprn2	1.29	<0.01	protein tyrosine phosphatase receptor type N2	Signaling receptor; potential pancreas-kidney axis influence on glucose handling.	[27]	D, I
Aldoc	0.7	<0.05	aldolase C	Glycolytic enzyme; energy metabolism in kidney cells; may modulate cellular response to hyperglycemia.		D, B12, I
Rph3a	1.35	<0.05	rabphilin 3A	Vesicle trafficking regulator; podocyte/vesicular transport implications.		D, B12, I
Wnt11	1.25	0.07	wingless-type MMTV integration site family member 11	Wnt signalling; implicated in repair/remodeling; could modulate maladaptive fibrosis in DN.	[23]	D
Cfap52	1.24	0.06	cilia and flagella associated protein 52	Ciliary function in tubular epithelial sensing; potential impact on tubular injury/repair.	[22]	B12
Col20a1	-0.81	<0.001	collagen, type XX, alpha 1	ECM collagen; may impact fibrosis and mesangial expansion.	[27]	D, B12, I
Ffar3	-1.13	0.07	free fatty acid receptor 3 (GPR41)	Fatty-acid sensing receptor; may affect kidney inflammation/metabolism		D, B12
Ttn	-0.91	<0.0001	titin	Large cytoskeletal protein; potential mechanosensing role in glomerular cells	[27]	D, B12, I
Muc6	-0.9	<0.01	mucin 6	Secreted mucin; possible deposition/ECM interactions in injury		B12, I
Slc22a4	0.2	<0.01	solute carrier family 22 member 4 (OCTN1)	Organic cation transporter; may modify tubular handling of metabolites/drugs		D, B12, I
**Gene**	**log2FC**	***Padj***	**Full gene name**	**Role in diabetic nephropathy**	**Reference**	**Effect**

Aqp6	0.8	<0.0001	aquaporin 6	Aquaporin water channel; may modulate renal water handling under diabetic conditions.		D, B12, I
Slc4a1	0.46	<0.01	solute carrier family 4 (anion exchanger) member 1 (AE1)	Anion exchanger; kidney/collecting duct roles could impact acid–base and tubular function.		D,B12, I
Slc44a3	0.48	<0.01	solute carrier family 44 member 3	Choline-like transporter; DN role speculative.		
Slc10a5	-0.35	0.11	solute carrier family 10 member 5	Bile acid or related transporter family member; DN role speculative.		D, B12
Slc25a19	-0.38	<0.05	solute carrier family 25 member 19	Mitochondrial thiamine pyrophosphate transporter; mitochondrial metabolism relevance.		B12, D
Slc4a5	0.99	<0.05	solute carrier family 4 member 5 (NBCe2)	Sodium-bicarbonate transporter: genetic variants linked to BP regulation is a major DN modifier.		B12
Txnip	0.89	<0.0001	thioredoxin interacting protein (TXNIP)	Mediates oxidative stress, inflammasome activation and glucose-induced damage; strongly implicated in DN pathogenesis.	[21, 28]	D, B12, I
Gpx6	0.28	0.44	glutathione peroxidase 6	Antioxidant enzyme family member; potential oxidative stress defense.		D
Maoa	0.28	0.07	monoamine oxidase A	Oxidative enzyme; may influence ROS/oxidative stress in renal tissue.		B12
Pex5	0.17	0.12	peroxisomal biogenesis factor 5	Peroxisome function; lipid oxidation and ROS handling in kidney.		D, B12,I
Txnrd3	0.26	<0.05	thioredoxin reductase 3	Mitochondrial redox enzymes: inferred mitochondrial oxidative stress role in DN.		B12
Bmal1	-1.65	<0.01	basic helix-loop-helix ARNT like 1 (ARNTL)	Core clock TF. Disruption worsens metabolic stress, inflammation and renal injury in DN models.	[32, 33, 38]	B12
Npas2	-1.41	0.06	neuronal PAS domain protein 2	Clock TF — supports circadian regulation of renal metabolism; inferred DN relevance.	[32, 33]	B12
Clock	-0.32	0.07	clock circadian regulator	Core clock gene; circadian disruption influences renal metabolism, blood pressure, inflammation in DN.	[32, 33, 39]	D, B12
Per2	0.32	0.3	period circadian clock 2	Clock output; maintains rhythmic gene expression and metabolic homeostasis; dysregulation can worsen DN phenotypes.	[32, 33]	D, B12
Cry1	0.59	0.12	cryptochrome circadian regulator 1	Maintains circadian feedback and metabolic stability	[32, 33]	B12
Per3	0.69	<0.05	period circadian clock 3	Contributes to rhythmic expression of renal metabolic genes.	[32, 33]	B12
**Gene**	**log2FC**	***Padj***	**Full gene name**	**Role in diabetic nephropathy**	**Reference**	**Effect**

Per1	0.7	<0.01	period circadian clock 1	Important in renal rhythmic gene regulation and sodium handling; affects BP and DN progression.	[32, 33, 40]	B12
Cry2	0.73	<0.05	cryptochrome circadian regulator 2	Clock protein with metabolic regulation roles; inferred DN relevance via NAD+ and metabolic pathways.	[32, 33]	B12
Nampt	0.42	<0.01	nicotinamide phosphoribosyltransferase	Regulates NAD+ salvage and links circadian clock to SIRT1/energy metabolism; implicated in diabetic metabolic stress; DN relevance via metabolism and inflammation.	[24, 41]	B12
H1f0	0.57	<0.0001	H1.0 linker histone	Chromatin compaction; epigenetic regulation under hyperglycemia; inferred involvement in persistent transcriptional changes in DN.	[44, 45, 46]	D,B12, I
H1f2	0.73	<0.001	H1.2 linker histone, cluster member	Linker histone; epigenetic regulator; DN role inferred from hyperglycemia-driven chromatin changes.	[44, 45, 46]	B12, I
H1f10	1.09	<0.05	H1.10 linker histone	Epigenetic control under chronic hyperglycemia.	[44, 45, 46]	I
H1f3	1.12	<0.01	H1.3 linker histone	Chromatin regulation; inferred link to persistent transcriptional changes in DN.	[44, 45, 46]	I
Wee1	0.52	<0.05	WEE1 G2 checkpoint kinase	prevents abnormal cell cycle reentry and podocyte damage in DN	[25]	D, B12
Cdk20	-0.57	<0.01	Cyclin-dependent kinase 20	Could be relevant in kidney epithelial cell proliferation or injury response	[26]	
