# Supplementary figures and images for: Vitamin B12 Protects Against Early Diabetic Kidney Injury and Alters Clock Gene Expression in Mice
Source: Biomolecules. 2025 Dec 3;15(12):1689. doi: 10.3390/biom15121689 (PMC12731228; doi:10.3390/biom15121689)

Trial #1

Bmal1 *and* bActin

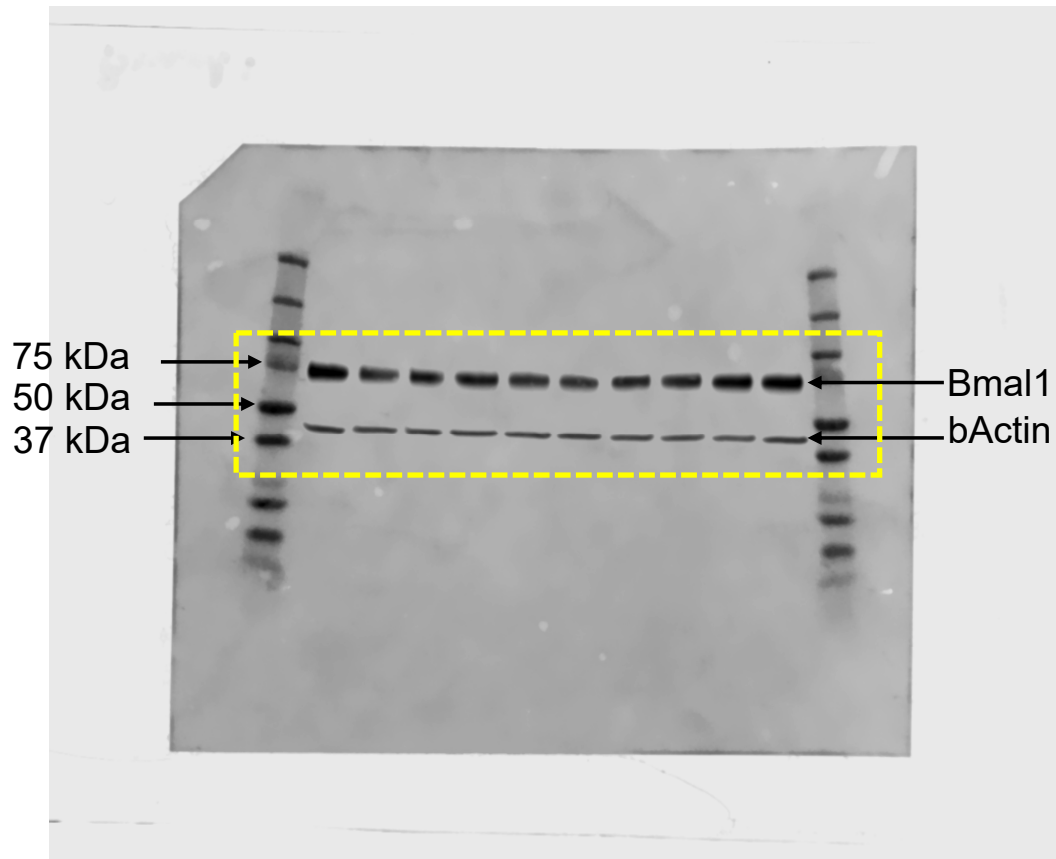

Per1

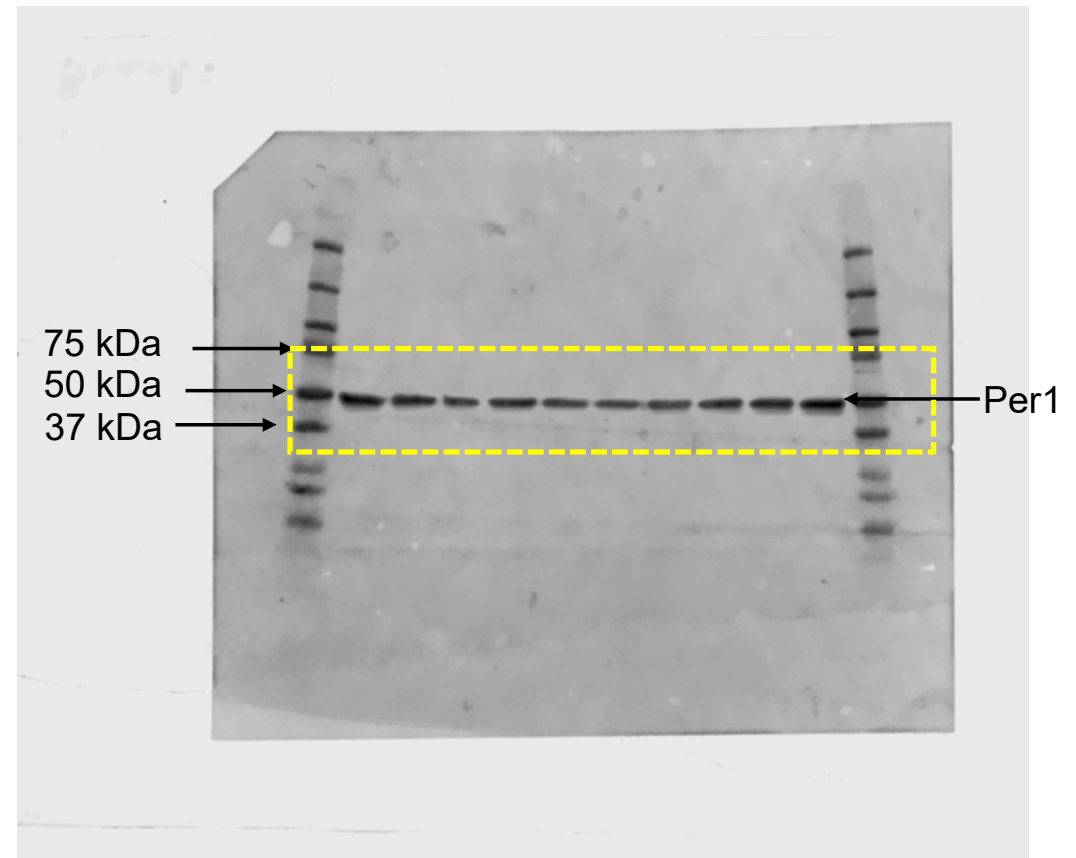

Trial #2

Bmal1 *and* bActin

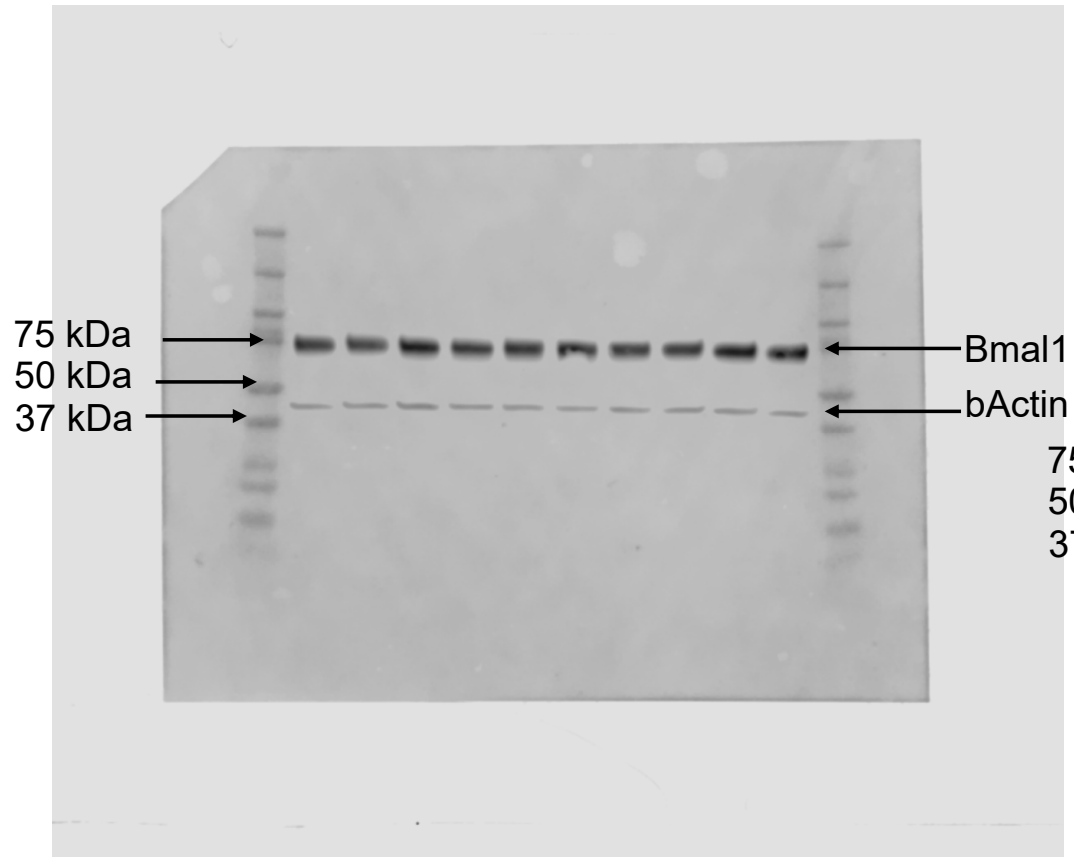

Per1 *and* bActin

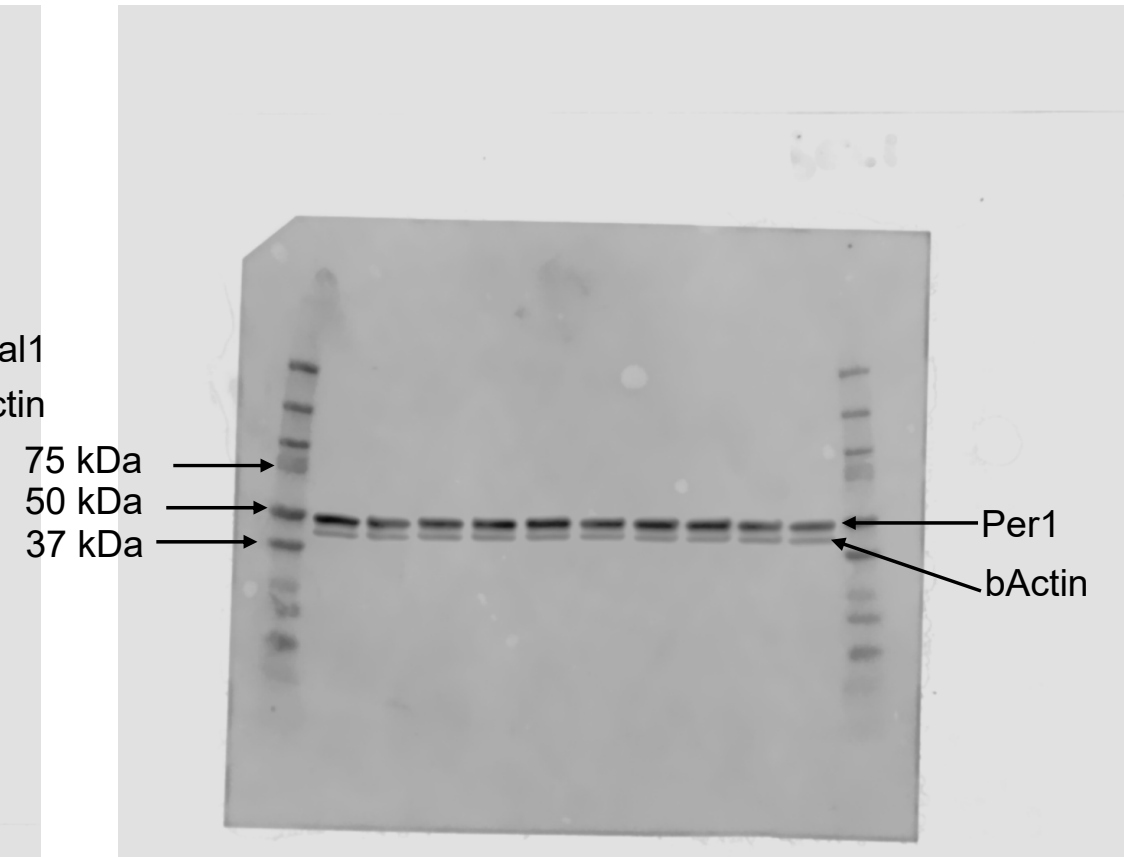

Trial #3

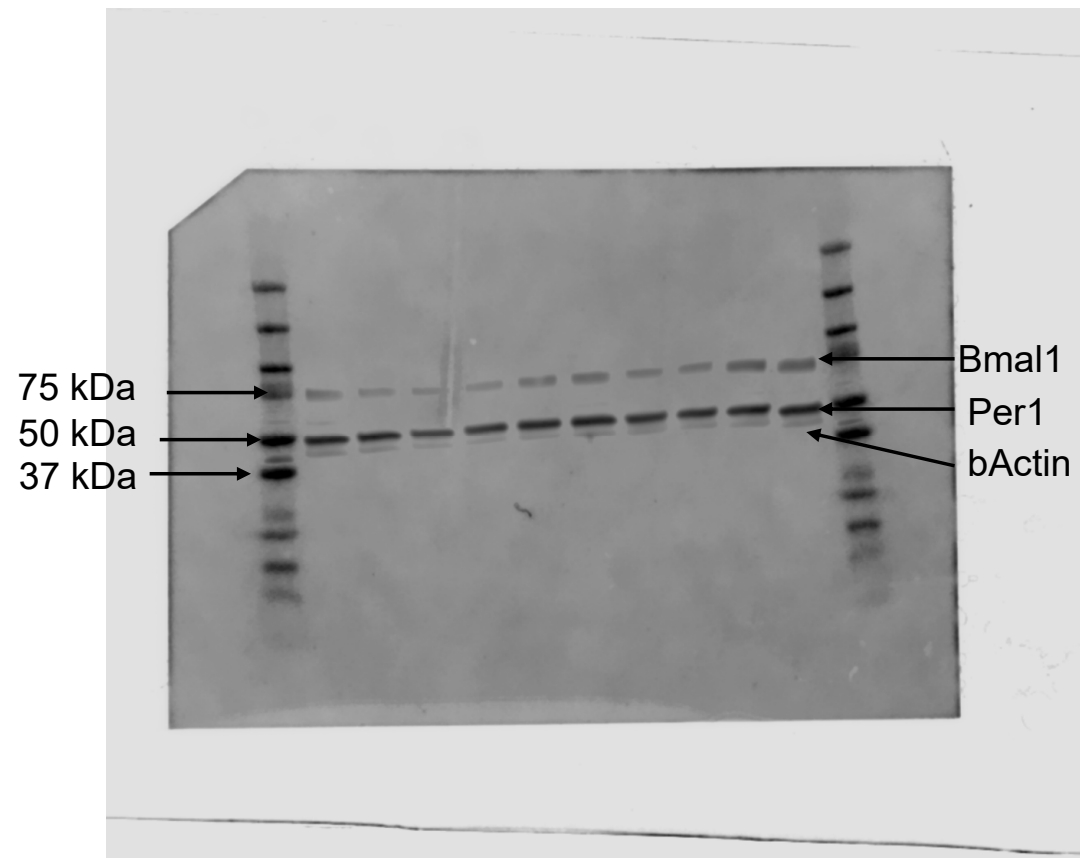

Selected from Trial #1:

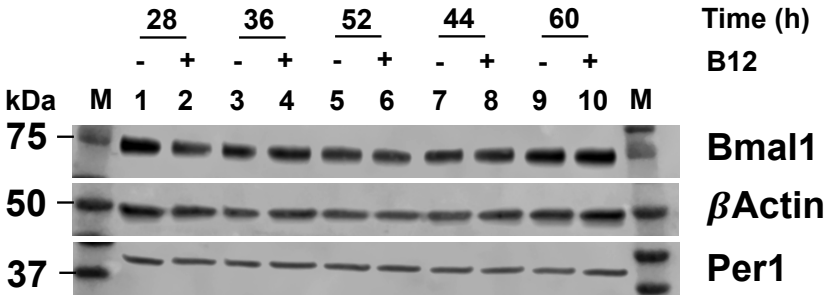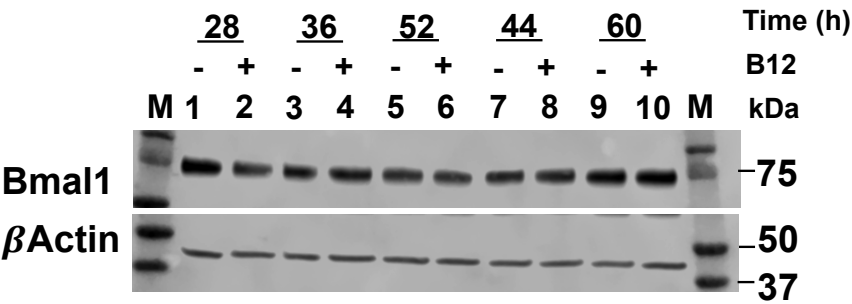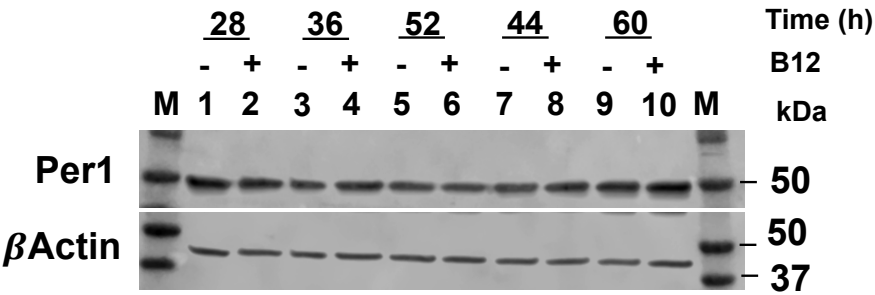

Supplement: Supplementary file 1 [file biomolecules-15-01689-s001.zip › Original images for western blot.pdf]
